# The Purinergic Receptor P2rx3 is Required for Spiral Ganglion Neuron Branch Refinement during Development

**DOI:** 10.1523/ENEURO.0179-20.2020

**Published:** 2020-08-07

**Authors:** Zhirong Wang, Johnny S. Jung, Talya C. Inbar, Katherine M. Rangoussis, Christian Faaborg-Andersen, Thomas M. Coate

**Affiliations:** Department of Biology, Georgetown University, Washington, DC 20007

**Keywords:** auditory, cochlea, hair cell, purinergic, spiral ganglion, synapse formation

## Abstract

The mammalian cochlea undergoes a highly dynamic process of growth and innervation during development. This process includes spiral ganglion neuron (SGN) branch refinement, a process whereby Type I SGNs undergo a phase of “debranching” before forming unramified synaptic contacts with inner hair cells. Using *Sox2*^CreERT2^ and *R26R*^tdTomato^ as a strategy to genetically label individual SGNs in mice of both sexes, we report on both a time course of SGN branch refinement and a role for P2rx3 in this process. P2rx3 is an ionotropic ATP receptor that was recently implicated in outer hair cell spontaneous activity and Type II SGN synapse development (Ceriani et al., 2019), but its function in Type I SGN development is unknown. Here, we demonstrate that P2rx3 is expressed by Type I SGNs and hair cells during developmental periods that coincide with SGN branching refinement. *P2rx3* null mice show SGNs with more complex branching patterns on their peripheral synaptic terminals and near their cell bodies around the time of birth. Loss of *P2rx3* does not appear to confer general changes in axon outgrowth or hair cell formation, and alterations in branching complexity appear to mostly recover by postnatal day (P)6. However, when we examined the distribution of Type I SGN subtypes using antibodies that bind Calb2, Calb1, and Pou4f1, we found that *P2rx3* null mice showed an increased proportion of SGNs that express Calb2. These data suggest P2rx3 may be necessary for normal Type I SGN differentiation in addition to serving a role in branch refinement.

## Significance Statement

P2rx3 receptors are a class of ionotropic purinergic receptors that are expressed in sensory afferent neurons and have been shown to play essential roles in sensory transduction. However, little is known about how P2rx3 functions in neuronal morphogenesis and synaptic connectivity. Here, we found that P2rx3 is expressed by spiral ganglion neurons (SGNs) and hair cells during cochlear development. Using *P2rx3* null mice combined with genetic sparse labeling, we discovered P2rx3 regulates SGN branch refinement, which is a function of P2rx3 distinguishable from the more conventionally-known role in neural transduction. These results offer new insights into how P2rx3 promotes auditory neuron maturation, which may be useful for endeavors aimed at regenerating lost auditory connections in hearing loss.

## Introduction

Hearing function depends on the development and maintenance of spiral ganglion neurons (SGNs) and their precise patterns of wiring with sensory hair cells in the cochlea. SGNs are bipolar neurons that extend peripheral axons toward hair cells, and central axons into the brainstem as part of the VIIIth cranial nerve (Nayagam et al., 2011). Early in auditory development, SGN progenitors develop into either Type I or Type II SGNs, which innervate inner hair cells and outer hair cells respectively (Appler and Goodrich, 2011; Bulankina and Moser, 2012). Before establishing fully mature connections, the SGN peripheral axons undergo an array of dynamic developmental events including axon outgrowth, target selection, refinement, spontaneous activity, and pruning (Coate et al., 2019). In auditory transduction, glutamate is released from hair cells onto SGNs (Glowatzki and Fuchs, 2002) at ribbon-type synapses, which are formed, in rodent models, during early postnatal stages (Michanski et al., 2019). Recently, advances in single cell RNA sequencing technology helped reveal that Type I SGNs differentiate into three molecularly distinguishable subtypes (Petitpré et al., 2018; Shrestha et al., 2018; Sun et al., 2018), and that their differentiation is driven by synaptic activity (Shrestha et al., 2018; Sun et al., 2018). The subtype of each SGN likely defines its function and synaptic location on inner hair cells (Liberman, 1982; Wu et al., 2016; Sherrill et al., 2019).

The mechanisms that control neuronal morphogenesis and synapse formation are fundamental questions in developmental neurobiology (Luo, 2002). During development, both presynaptic and postsynaptic terminals sculpt their structures by eliminating excessive branches, which is a process of refinement critical for normal function of the nervous system (Gibson and Ma, 2011; Kalil and Dent, 2014; Riccomagno and Kolodkin, 2015; Schuldiner and Yaron, 2015). In the developing auditory system, each SGN extends a single peripheral axon that initially shows extraneous branches (Koundakjian et al., 2007) that are progressively lost as development progresses. Previously, it was shown that Semaphorin-5B/Plexin-A1 interactions are important for these events: Sema5B is expressed by hair cells while PlexinA1 is expressed by SGNs, and loss of either factor leads to more elaborate SGN branching patterns (Jung et al., 2019). Here, we report that signaling by P2rx3 serves a similar role.

ATP serves as the intracellular energy currency but also can be released into the extracellular space to act as a neurotransmitter. There are two large groups of membrane-bound purinergic receptors: the ionotropic P2X family, which includes seven family members, and the metabotropic P2Y family, which includes eight family members. P2X receptors are ATP-gated cation channels that allow sodium and calcium ions to flow into the cell (Khakh and North, 2006), whereas P2Y receptors transduce ATP signals via G-protein-mediated intracellular signaling pathways (Burnstock, 2006). Intracellular calcium increases result from purinergic receptor activation leading to a variety of signaling responses (Khakh and North, 2012), with increases in neuronal excitability as the most commonly understood response (Burnstock, 2012). For example, in gustatory excitation, P2rx3 receptors are localized postsynaptically at junctions between sensory cells and taste afferents and they have been demonstrated to be the primary receptor for extracellular ATP (Finger et al., 2005). But, purinergic signaling is also known to be involved in a variety of aspects of nervous system development, including neuron proliferation, migration, maturation, differentiation, and survival (Zimmermann, 2011). Notably, P2X signaling has been shown to regulate the actin cytoskeleton in neurites by signaling through cofilin (Homma et al., 2008), suggesting extracellular ATP can regulate dynamic changes in neuronal architecture. In this study, we leveraged sparse neuron labeling techniques and found a novel role for P2rx3 in regulating Type I SGN branch refinement during cochlear development. We also found that P2rx3 is necessary for the development of the normal profile of Type I SGN subtypes.

## Materials and Methods

### Mouse lines

All animals in this study were maintained in accordance with the Georgetown University Institutional Animal Care and Use Committee (protocol #1147). Both male and female mice were used for all experiments. *P2rx3* null mice (Cockayne et al., 2000) were a kind gift from Thomas Finger at University of Colorado School of Medicine. *P2rx3* knock-out (KO) mice were bred and maintained onto a C57BL/6 background using breeder mice from Charles River Laboratories. *Sox2*^CreER^ and *R26R^tdTomato^* reporter mice were originally purchased from The Jackson Laboratory (Stocks No. 017593 and 007914). The *Atoh1*^nGFP^ allele (Lumpkin et al., 2003) was also maintained on this line so the positions of hair cells could be referenced. The primers used for genotyping are as follows: *P2rx3* common: AGT GGA GTT CTT GGC TCA GG, *P2rx3* wild-type (WT) reverse: GCT TTT CAC AAC CAC CGA CT, *P2rx3* mutant reverse: CCT TCT TGA CGA GTT CTT CTG AG. *Rosa26* forward: AAG GGA GCT GCA GTG GAG TA, *Rosa26* reverse: CCG AAA ATC TGT GGG AAG TC, *tdTomato* forward: GGC ATT AAA GCA GCG TAT CC, *tdTomato* reverse: CTG TTC CTG TAC GGC ATG G. *Sox2*^CreER^ WT forward: ACC AGC TCG CAG ACC TAC AT, *Sox2*^CreER^ WT reverse: CGG GGA GGT ACA TGC TGA T, *Sox2*^CreER^ mutant forward: CCA AAA ACT AAT CAC AAC AAT CGC, *Sox2*^CreER^ mutant reverse: GGC AAA CGG ACA GAA GCA T. In nearly all experiments, *P2rx3* heterozygous males and females were crossed to generate KOs and littermate controls (WT). Both left and right ears were used from one animal; typically, *N* (sample size) equals one cochlea, and *n* equals one SGN peripheral axon. For the synapse and neuronal subtype staining experiments, *P2rx3* WT breeding pairs and KO breeding pairs were set up separately and only one ear from one animal was used to generate more diverse biological replicates. In all cases, breeders were strain matched and experimental progeny were age matched. For timed pregnancies, plug dates were assumed to be embryonic day (E)0.5; the day of birth was considered as postnatal day (P)0.

### Immunohistochemistry and antibodies

To prepare tissues for immunostaining, whole heads with the brain removed were fixed in 4% paraformaldehyde (PFA) for 45 min at room temperature (RT) and rinsed extensively in 1× PBS. For synapse staining, tissues were fixed for 25 min in 4% PFA without prior exposure to PBS. For neuronal subtype staining, cochleae were perfused through the round window with 4% PFA without prior exposure to PBS then bath-fixed for 1 h at RT. Bony capsules were decalcified with 0.5 m EDTA for 24 h. For whole-mount preparations, cochlear capsules, stria vascularis, and Reissner’s membranes were removed before staining in glass vials. For cross-sections, inner ears were stepped through 10%, 20%, and 30% sucrose and then embedded and frozen in optimal cutting temperature (OCT; Sakura Finetek) and sectioned at 12 μm. For most staining experiments, primary antibodies were applied at 4°C overnight and secondary antibodies were applied at RT for 1 h. For synapse staining, primary antibodies were applied overnight at 37°C; secondary antibodies were applied overnight at 4°C. For neuronal subtype staining, sections underwent antigen retrieval before immunostaining, which was adopted from (Sherrill et al., 2019). Slides were suspended over boiling water in a steamer, and sodium citrate buffer (10 mm sodium citrate and 0.05% Tween, pH 6.0) was added to cover the surface of each slide. Slides with buffer were steamed for 30 min then cooled at RT for 5 min. They were then rinsed in PBS a minimum of 10 min. For immunostaining that followed, primary antibodies were added to sections overnight at 37°C. Fluorescent secondary antibodies (1:1000) were added to the samples for 1 h.

The antibodies and concentrations used in this paper were as follows: rabbit-anti-GAP43 (Millipore AB5220, 1:1000, RRID: AB_2107282), goat-anti-Sox10 (R&D System AF2864, 1:500, RRID: AB_442208), rabbit-anti-dsRed (Clontech 632496, 1:2000, RRID: AB_10013483), mouse-anti-Tuj1 (Biolegend, 1:1000, RRID: AB_2313773), rabbit-anti-MyosinVI (Proteus Biosciences 25–6791, 1:1000, RRID: AB_10013626), goat-anti-MyosinVI (Coate et al., 2015; 1:1000, RRID: AB_2783873), goat-anti-Sox2 (R&D Systems AF2018, 1:500, RRID: AB_355110), chicken-anti-neurofilament heavy chain (NFH or NF200; Aves Labs, 1:1000, RRID: AB_2313552), rabbit-anti-P2rx3 (Alomone Labs APR-016, 1:1000, RRID: AB_2313760), goat-anti-ribeye (Santa Cruz Biotechnology SC-5967, 1:500, RRID: AB_2086771), rabbit-anti-shank1a (Neuromics PA19016, 1:1000, RRID: AB_1622814), chicken-anti-Calb1 (Abeomics 34–1020, 1:500, RRID: AB_2810884), rabbit-anti-Calb2 (Thermo Fisher Scientific PA5-34 688, 1:1000, RRID: AB_2552040), and mouse-anti-Pou4f1 (Millipore Sigma MAB1585, 1:100, RRID: AB_94166). Actin stereocilia bundles were detected by 405-phalloidin conjugate at 1:1000 (Santa Cruz Biotechnology), cell nuclei were detected by DAPI at 1 μg/ml (Santa Cruz Biotechnology). Host-specific 488 Alexa Fluor, Cy3, and Cy5 secondary antibodies were used (1:500) accordingly.

### Experimental design and statistical analyses

All statistical tests were performed using Prism 8.0 (GraphPad). Results were reported as mean ± SEM. A two-tailed unpaired *t* test with Welch’s correction was used to determine statistical significance unless specifically noted; *p* ≥ 0.05, ns; *p < 0.05; **p < 0.01, ***p < 0.001, ****p < 0.0001. Please see figure legends for statistical tests and sample sizes.

To quantify cochlear length, measurement lines (in Fiji) were drawn along the region of the inner pillar cells from the apex to the base; 25% of cochlear length was defined as the apical region, 50% as the middle region, and 75% as the basal region for position-matched comparisons. To quantify radial bundle length using NF200-stained whole-mount samples, line length measurements were taken (eight per region of each sample) along the extending radial bundles and then averaged.

Quantifications of sparsely labeled SGN terminals and cell bodies were performed using the filament function of Imaris (Bitplane). High-magnification images were taken using a Zeiss laser scanning confocal microscope (LSM 880) through a 63× objective (Plan-Apochromat 63×/1.40 Oil DIC M27) at 1024 by 1024 pixels. Z-stacks stepped by optimal 0.42 μm were adjusted in individual images to include the entire range of terminal arborization. Only non-overlapping terminals were quantified. For reconstructing axonal terminals, the filament starting point was determined based on the first branching point near the inner hair cells. The entire terminal arborization was traced by drawing terminal points. For each neuron, a skeleton fitting the center of the fluorescence intensity and branching points was determined automatically by the software. The reconstructed filament was then manually centered and smoothed once before finishing the final rendering. For the P0 terminal analyses, the terminal ending position of each axon was scored as modiolar, pillar, or ambiguous. For the single-neuron analyses, *n* (sample size) is equal to one axon. Depending on the number of collateral branches, multiple starting points were drawn whenever a branch originated from the main axon.

Quantification of synaptic structures was performed using the surface and spot functions of Imaris. Background subtraction and thresholding was applied uniformly across all samples. For reconstructing Shank1a patches, the Split Touching Objects function was enabled by a constant seed point’s diameter to ensure single hair cell reconstruction. Minute values were either deleted as noise or unified to the adjacent hair cell by a cutoff surface area of 1 μm. For reconstructing ribeye puncta, the Different Spot Sizes (region growing) function was enabled. Estimated XYZ diameter was determined empirically and kept consistent. Absolute intensity was used for spot region type and region border was used to determine the final rendering.

Type I SGN subtype immunostaining of WT and *P2rx3* KO samples was quantified (blinded to genotype) using ImageJ. A maximum intensity projection was made from images captured with a 20× objective (Plan-Apochromat 20×/0.8 M27); triple-immunostaining for Pou4f1, Calb1, Calb2 plus DAPI was visualized in four separate channels. To control for any sample-to-sample variation in background levels, pixel intensities were examined on non-SGNs (see arrowheads in 9I-L; possibly Schwann or otic mesenchyme cells). SGNs with strong staining above this value were deemed positive. Pou4f1-positive cells were determined by obvious nuclear staining above background levels. The DAPI channel was used to confirm overlap of Pou4f1 staining with nuclei. We note here that all SGNs show Pou4f1 background staining in the cytoplasm. Anti-Calb2 and anti-Calb1 immunostaining leads to strong signal in both the nucleus and cytoplasm; pixel values of nearby non-SGNs were also used to determine background in these channels. In terms of the workflow, we first determined which SGNs were positive for Pou4f1. We then toggled between channels to determine whether each was also positive for Calb1 or Calb2. Following this, Calb2 and Calb1 single-positive neurons were counted. Finally, overlap between Calb1 and Calb2-positive cells was counted by toggling between channels. Each SGN was annotated along the way to ensure it was not counted twice. Following quantifications, polygons were drawn around SGN bundles to measure the SGN area. SGN density was calculated by dividing total number of cell bodies by the SGN area.

## Results

### SGN branching refinement occurs during development

**Figure 1.**
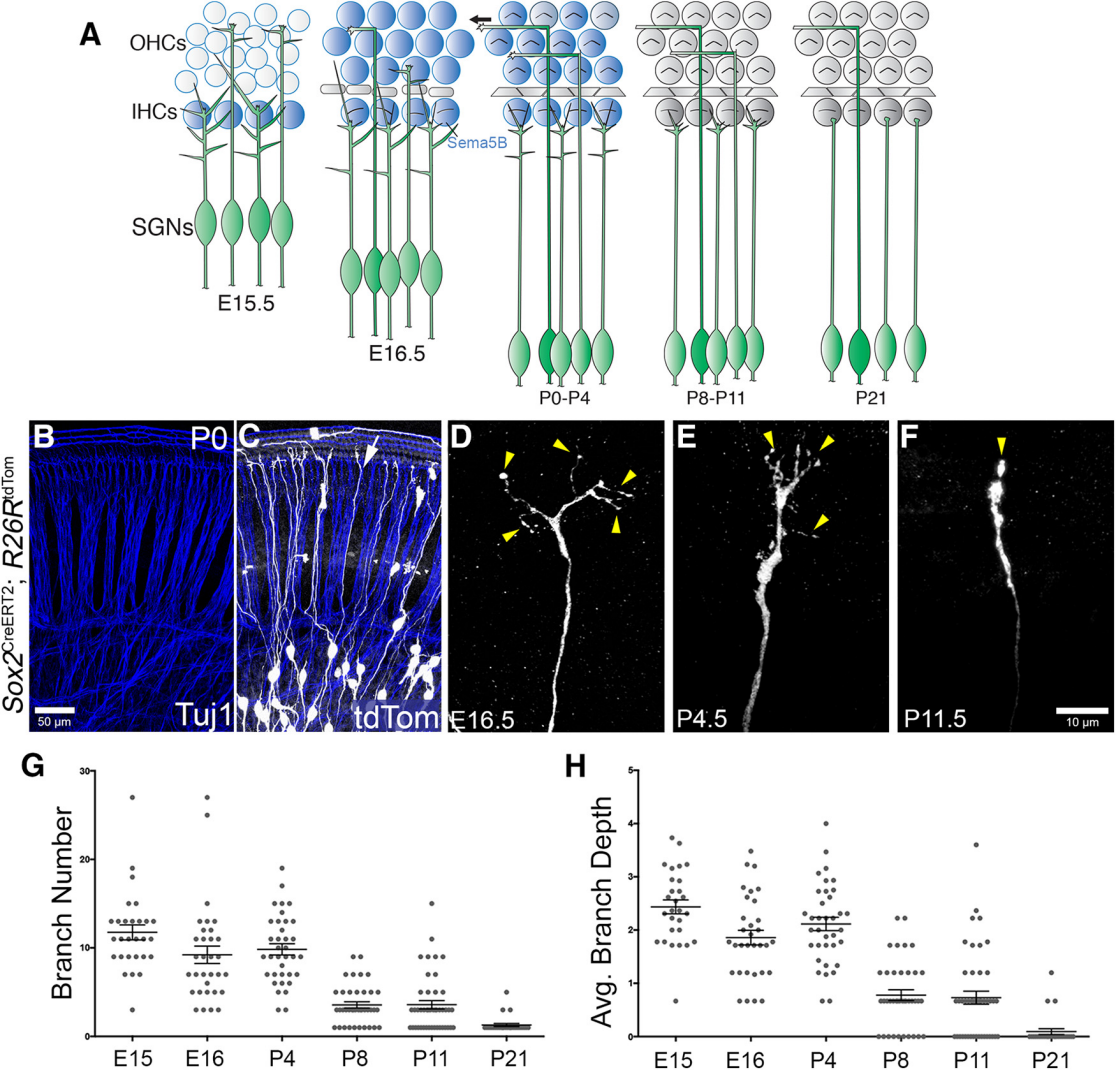
A developmental time course of SGN peripheral axonal refinement. ***A***, Cartoon schematic showing the approximate time points of SGN maturation. During the third and the last embryonic week of mouse cochlear development, SGNs extend numerous processes to interact with cells in the sensory epithelium. At the end of this week, Type I and Type II SGNs establish contacts with inner hair cells and outer hair cells, respectively. SGNs refine their terminal arborization as they mature and Type I SGN terminals form single connections with IHCs after the first postnatal week. Hair cells between E15.5 and P4 are shaded blue to indicate the period of Semaphorin-5B expression. IHC, inner hair cell; OHC, outer hair cell. E, embryonic day; P, postnatal day. ***B***, ***C***, Examples of sparse labeling strategy using *Sox2*^CreERT2^*; R26R*^tdTom^ to label a subset of neurons (white) in contrast to all neurons labeled by Tuj1 (blue). The white arrow points to a Type I SGN terminal. ***D–F***, Individual sparsely labeled Type I SGN terminal arborizations at three distinct time points throughout development. Yellow arrowheads point to terminal endings which become refined over time. ***G***, Scatter plots of individual axonal terminals quantified to show the number of individual branches within each terminal arborization which are reduced over time (in μm, E15.5, 11.8 ± 0.9, *n* = 28, *N* = 4; E16.5, 9.2 ± 1.0, *n* = 32, *N* = 3; and P4, 9.8 ± 0.6, *n* = 36, *N* = 6; P8, 3.6 ± 0.4, *n* = 39, *N* = 4; P11, 3.6 ± 0.5, *n* = 45, *N* = 5; P21, 1.3 ± 0.2, *n* = 27, *N* = 5; one-way ANOVA followed by Tukey’s multiple comparisons test). ***H***, Scatter plots of individual axonal terminals quantified to show the complexity of each terminal arborization which are reduced over time (E15.5, 2.4 ± 0.1; E16.5, 1.9 ± 0.1; and P4, 2.1 ± 0.1; P8, 0.8 ± 0.1; P11, 0.7 ± 0.1; P21, 0.09 ± 0.06; same samples and statistical tests as in ***G***). See Tables 1, 2 for detailed comparisons and results of statistical tests.

**Figure 2.**
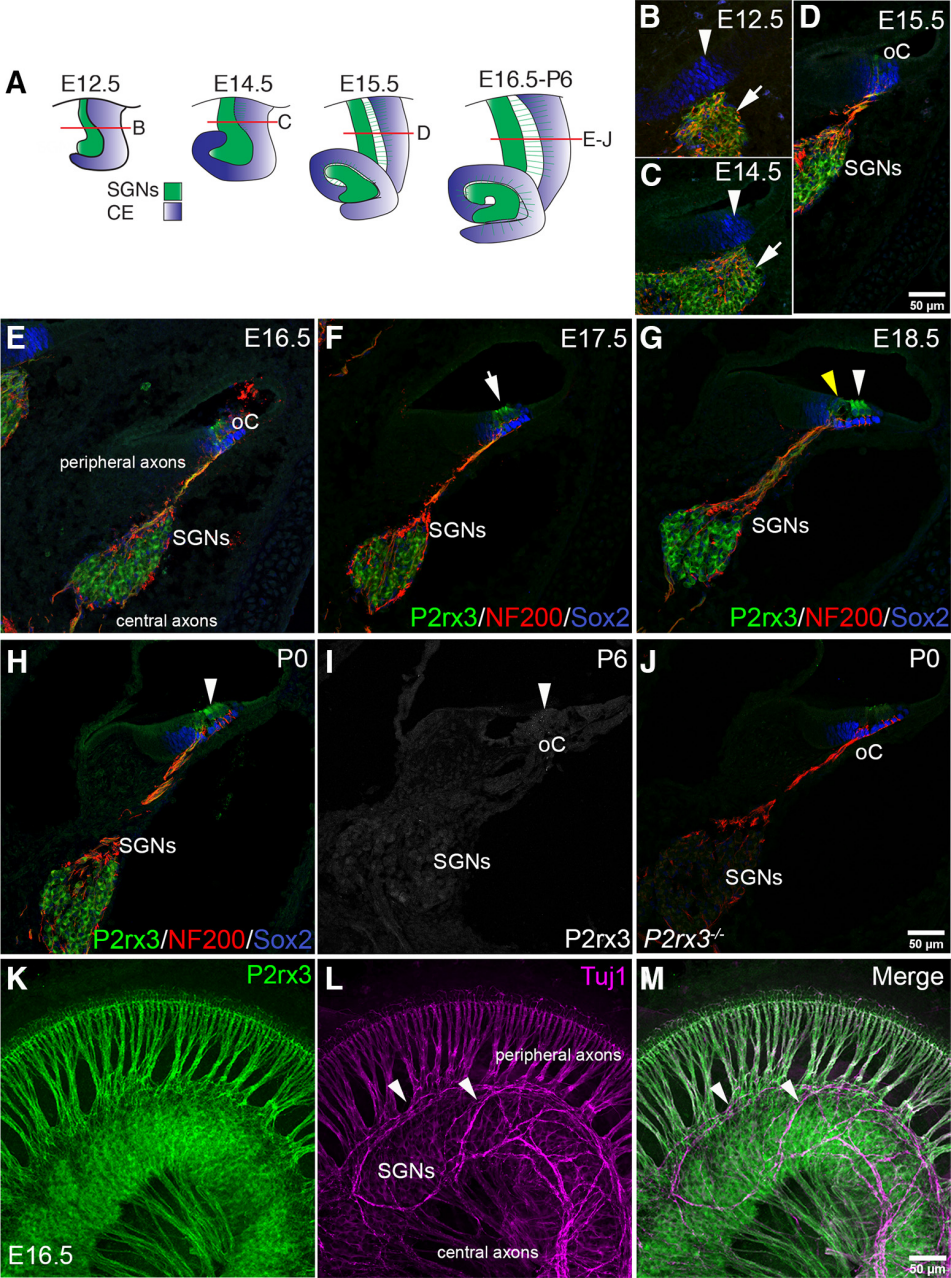
Expression of P2rx3 in SGNs and hair cells coincides with SGN branch refinement. ***A***, Cartoon schematic showing the developing mouse cochlea extending and coiling from E12.5 to P6. Green indicates SGNs and blue indicates cochlear epithelium. ***B***, ***C***, Cross-section view of E12.5–E14.5, SGNs (white arrows) are closely attached to the epithelium (white arrowhead) as they delaminate. P2rx3 immunostaining (green) is shown in SGNs; Sox2 (blue) shows the cochlear sensory domain and NFH (red) shows the SGN peripheral axons. ***D***, At E15.5, as SGNs extend peripheral axons to the organ of Corti (oC), P2rx3 is present on SGN cell bodies, peripheral axons, and newly differentiated inner hair cells. ***E***, ***F***, At E16.5 and E17.5, SGNs continue to express P2rx3, and as outer hair cells are differentiated, P2rx3 signals appear in outer hair cells (white arrow in ***F***) in addition to the inner hair cells. ***G***, ***H***, At E18.5 and P0, P2rx3 begins to disappear from the inner hair cells (yellow arrowhead in ***G***) while being maintained in all SGNs. The white arrowheads in ***G–I*** point to outer hair cells. ***I***, P2rx3 immunostaining is mostly absent at P6 in SGNs and HCs. ***J***, A cross-section from a P0 *P2rx3*−/− cochlea demonstrates complete absence of P2rx3 immunostaining. ***K–M***, A whole-mount view of an E16.5 cochlea showing P2rx3 present on SGN cell bodies, radial bundles, and axonal terminals; note that P2rx3 antibodies do not overlap with the Tuj1 staining (magenta) of the olivocochlear efferent fibers, which are prominent in the IGSB (white arrowheads).

Before innervating inner hair cells during cochlear development, Type I SGNs undergo a process of branch refinement (or “debranching”; Fig. 1*A*) that is dependent, in part, on Semaphorin-5B (Jung et al., 2019). We first wanted to determine the temporal progression of SGN debranching during development and did so by examining cochleae from mice carrying *Sox2*^CreERT2^ (Arnold et al., 2011) and *R26R*^tdTom^ (Madisen et al., 2010). As shown previously (Brooks et al., 2020), without tamoxifen treatment, low levels of Cre activity in these mice leads to tdTomato expression in various cell types such as SGNs, hair cells, Deiters’ cells, and glial cells. The resulting labeling is reliably sparse enough such that we can visualize individual SGN terminals clearly (Fig. 1*B*,*C*, arrow). Cochleae were collected at E15.5, E16.5, P4, P8, P11, and P21 (examples shown in Fig. 1*D–F*). A total of 30–40 SGN peripheral axons from the base for each stage were analyzed using Imaris. As expected, embryonic SGN peripheral axons show high branch number values (Fig. 1*G*) and branch depth values (Fig. 1*H*). “Branch depth” values were assigned based on the branching order: primary branch as 1, secondary as 2, tertiary as 3, etc. Average branch depth was calculated by the sum of each individual branch depth value divided by branch number; the higher branch depth value a particular neuron has, the more complex the terminal arborization. Interestingly, both branch number (ranging between 9 and 12) and depth values (ranging between 1.9 and 2.4) were quite stable between E15.5 and P4. At P8, SGN fibers showed a more narrowed appearance and significantly reduced branch number (3.6 ± 0.4, *n* = 39) and branch depth values (0.8 ± 0.1, *n* = 39; see Tables 1, 2 for statistical comparisons). By P21, the SGNs showed branch number and depth values that suggest a single contact with inner hair cells (branch number, 1.3 ± 0.2, *n* = 27; branch depth, 0.09 ± 0.06, *n* = 27). Many SGNs with single contacts were visible also between P8 and P11 (Fig. 1*F*). Together, SGN terminal branches clearly refine over the course of a four-week period. From E15.5 to P4, SGN branching values are stable, although this period involves active SGN motility (Coate et al., 2015). Following this period, from P4 to P8, there is a drastic decrease in branch number and complexity. The early phase correlates with the high levels of P2rx3 receptor expression in SGNs, suggesting a potential role of this ATP-gated ion channel in regulating SGN peripheral axon branching behavior (Fig. 2).

**Table 1:** Detailed comparisons of SGN branch numbers at different stages

Branch number Mean ± SEM	E15 11.8 ± 0.9	E16 9.2 ± 1.0	P4 9.8 ± 0.6	P8 3.6 ± 0.4	P11 3.6 ± 0.5	P21 1.3 ± 0.2
E15 11.8 ± 0.9	-	-	-	-	-	-
E16 9.2 ± 1.0	0.0845 n.s. [−0.19, 5.25]	-	-	-	-	-
P4 9.8 ± 0.6	0.3011 n.s. [−0.73, 4.57]	0.9827 n.s. [−3.17, 1.94]	-	-	-	-
P8 3.6 ± 0.4	<0.0001 **** [5.58, 10.79]	<0.0001 **** [3.15, 8.16]	<0.0001 **** [3.84, 8.7]	-	-	-
P11 3.6 ±0.5	<0.0001 **** [5.64, 10.7]	<0.0001 **** [3.21, 8.07]	<0.0001 **** [3.9, 8.61]	>0.9999 n.s. [−2.31, 2.29]	-	-
P21 1.3 ± 0.2	<0.0001 **** [7.62, 13.29]	<0.0001 **** [5.18, 10.67]	<0.0001 **** [5.86, 11.21]	0.1353 n.s. [−0.36, 4.9]	0.1109 n.s. [−0.28, 4.84]	-

In the conjunction cell where different stages in the column and the row meet, the first row indicates *p* values, the second row indicates the designation of statistical significance, the third row indicates 95% confidence intervals. These data show that branch numbers of developing SGNs decrease over time. n.s., not significant. ****p < 0.0001.

**Table 2:** Detailed comparisons of SGN branch depth at different stages

Average branch depth Mean ± SEM	E15 2.4 ± 0.1	E16 1.9 ± 0.1	P4 2.1 ± 0.1	P8 0.8 ± 0.1	P11 0.7 ± 0.1	P21 0.09 ± 0.06
E15 2.4 ± 0.1	-	-	-	-	-	-
E16 1.9 ± 0.1	0.02 * [0.057, 1.1]	-	-	-	-	-
P4 2.1 ± 0.1	0.4518 n.s. [−0.19, 0.83]	0.659 n.s. [−0.74, 0.23]	-	-	-	-
P8 0.8 ± 0.1	<0.0001 **** [1.16, 2.15]	<0.0001 **** [0.6, 1.56]	<0.0001 **** [0.87, 1.8]	-	-	-
P11 0.7 ± 0.1	<0.0001 **** [1.22, 2.19]	<0.0001 **** [0.66, 1.59]	<0.0001 **** [0.93, 1.83]	0.9996 n.s. [−0.39, 0.49]	-	-
P21 0.09 ± 0.06	<0.0001 **** [1.8, 2.88]	<0.0001 **** [1.24, 2.29]	<0.0001 **** [1.51, 2.53]	0.0017 ** [0.18, 1.19]	0.0032 ** [0.15, 1.13]	-

In the conjunction cell where different stages in the column and the row meet, the first row indicates *p* values, the second row indicates the designation of statistical significance, the third row indicates 95% confidence intervals. These data showed that branch depth of developing SGNs decreases over time. n.s., not significant. *p < 0.05; **p < 0.01; ****p < 0.0001

### P2rx3 is expressed by SGNs and hair cells during cochlear innervation

Purinergic signaling has been implicated in numerous biological functions, but little is known about its involvement in the developing cochlea. Previously, P2rx3 protein was shown to be expressed by mouse SGNs and hair cells in the cochlea around the time of birth (Huang et al., 2006). In addition, online databases show *P2rx3* mRNA expressed by SGNs as early as E12.5, and absent after P6 (Visel et al., 2004; Lu et al., 2011; Li et al., 2020), and in hair cells from E16.5 to P3 (Burns et al., 2015; Cai et al., 2015; Elkon et al., 2015; Scheffer et al., 2015). To examine P2rx3 distribution in the developing cochlea, we performed anti-P2rx3 antibody staining on cochlear cross-sections from a series of developmental stages (Fig. 2*A*). For all stages examined, the samples were counterstained with anti-Sox2 antibodies to show the position of the developing sensory domain (Kiernan et al., 2005) and anti-NF200 antibodies to show the position of the SGNs. At E12.5 and E14.5, P2rx3 protein is clearly detectable on SGNs but is not detectable on cells within the sensory domain (Fig. 2*B*,*C*). By E15.5, developing inner hair cells show P2rx3 protein at faintly detectable levels (Fig. 2*D*,*E*, oC), while SGNs show P2rx3 at robust levels (Fig. 2*D*). At E16.5 and E17.5, P2rx3 receptors are visible on SGNs and all hair cells (Fig. 2*E*,*F*); this pattern lasts only through E18.5 when the P2rx3 signal disappears from the inner hair cell (Fig. 2*G*, yellow arrowhead). By P0, P2rx3 expression on outer hair cells becomes minimal (Fig. 2*H*) and by P6 at the middle turn of the cochlea, neither SGNs nor hair cells show detectable levels of P2rx3 (Fig. 2*I*). The white arrowhead in G-I points to outer hair cells. We note here that, at the very apex of the cochlea at P6, P2rx3 protein was sometimes faintly visible (data not shown). This temporal pattern of protein expression aligns precisely with the pattern of *P2rx3* mRNA reported previously (Lu et al., 2011; Li et al., 2020). *P2rx3*−/− tissue showed no P2rx3 immunostaining in SGNs and very faint background staining in hair cells, indicating the antibody is specific (Fig. 2*J*). During the course of these studies, we co-labeled fixed cochleae with anti-P2rx3 antibodies and various neuronal markers to determine whether P2rx3 is expressed in olivocochlear efferent neurons (Maison et al., 2016) in addition to SGNs. Overall, P2rx3 does not appear to be expressed by efferents. As shown in Figure 2*K–M*, P2rx3 signal is absent from fibers within the intraganglionic spiral bundle (IGSB), which is mostly comprised of the efferent tracks of axons from both medial olivocochlear (MOC) and lateral olivocochlear (LOC) neurons (Frank and Goodrich, 2018).

### P2rx3 does not mediate SGN peripheral axon outgrowth or hair cell formation

**Figure 3.**
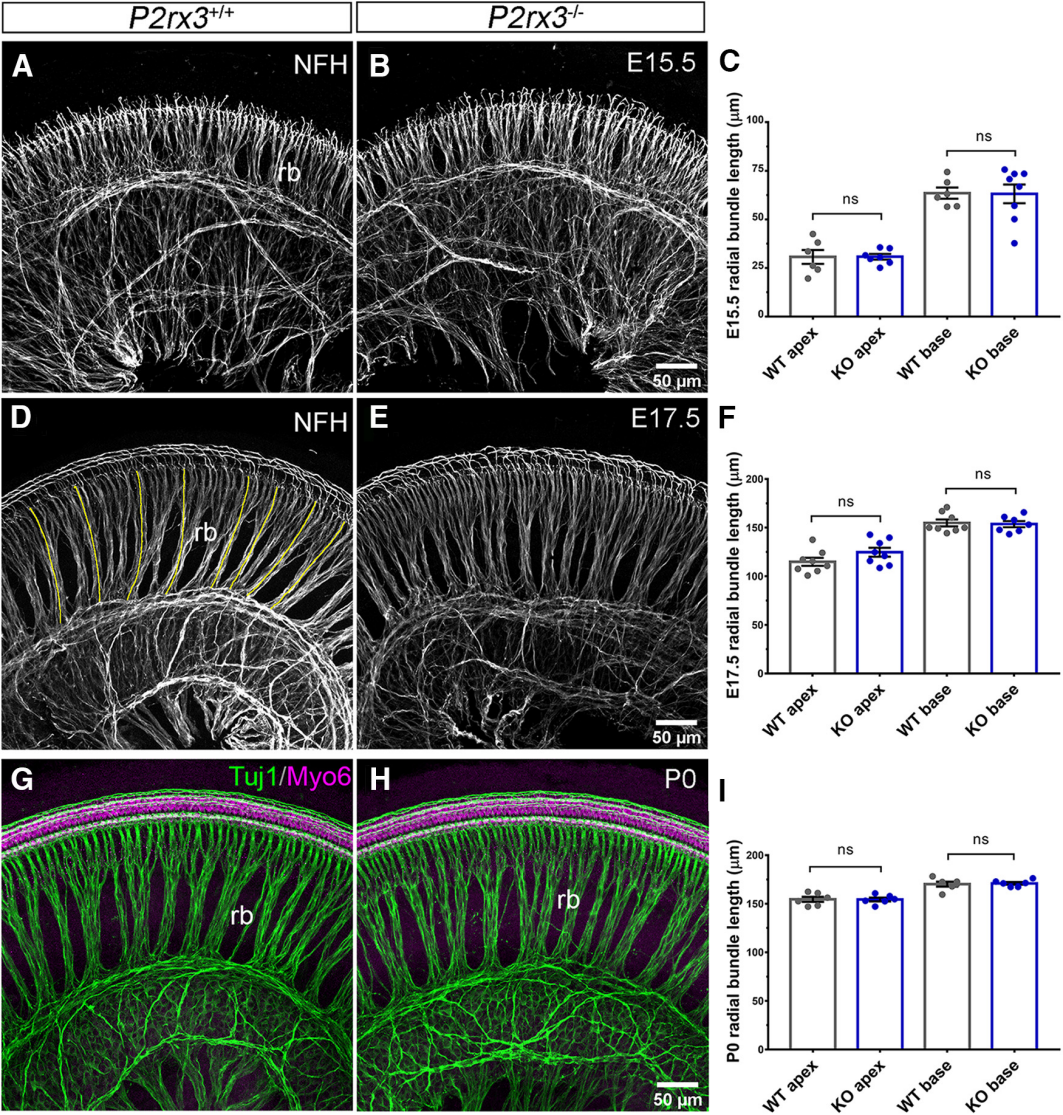
*P2rx3* is not required for SGN axonal outgrowth. ***A***, ***B***, WT and *P2rx3*−/− cochleae at E15.5 were labeled with NFH (gray) to reveal SGN peripheral axon radial bundles (rb). ***C***, Quantification of normalized radial bundle length at E15.5. Each datapoint represents one cochlea. *P2rx3*−/− cochleae show normal axonal outgrowth (in μm, WT apex: 30.7 ± 3.6, *N* = 6; KO apex: 30.8 ± 1.4, *N* = 7; WT base: 63.5 ± 2.9, *N* = 6; KO base: 63.1 ± 4.8, *N* = 8; four WT animals from two litters and four KO animals from two litters; comparison between WT and KO apex, *t*_(7)_ = 0.022, *p* = 0.983; comparison between WT and KO base: *t*_(11)_ = 0.071, *p* = 0.9444). ***D***, ***E***, Littermate control and *P2rx3*−/− cochleae at E17.5 were labeled with NFH (gray) to show SGN radial bundles. Yellow lines indicate radial bundle length. ***F***, Quantification of normalized radial bundle length at E17.5 and *P2rx3*−/− is comparable to WT littermates (in μm, WT apex: 114.9 ± 4.1, *N* = 8; KO apex: 124.7 ± 4.6, *N* = 8; WT base: 154.8 ± 3.5, *N* = 8; KO base: 153.6 ± 3.1, *N* = 7; four WT animals from four litters and four KO animals from four litters; comparison between WT and KO apex, *t*_(14)_ = 1.59, *p* = 0.1337; comparison between WT and KO base: *t*_(13)_ = 0.26, *p* = 0.7955). ***G***, ***H***, Littermate control and *P2rx3*−/− cochleae at P0 were labeled with NFH (green) to show SGN radial bundles and with Myo6 (magenta) to show HCs. ***I***, Quantification of normalized radial bundle length at P0. No axonal elongation defect was detected (in μm, WT apex: 154.6 ± 2.6, *N* = 6, KO apex: 154.4 ± 1.9, *N* = 6; WT base: 170.2 ± 2.6, *N* = 6; KO base: 171 ± 1.4, *N* = 6; three WT animals from three litters and three KO animals from three litters; comparison between WT and KO apex, *t*_(9)_ = 0.058, *p* = 0.9548; comparison between WT and KO base: *t*_(8)_ = 0.29, *p* = 0.7779). n.s., not significant.

**Figure 4.**
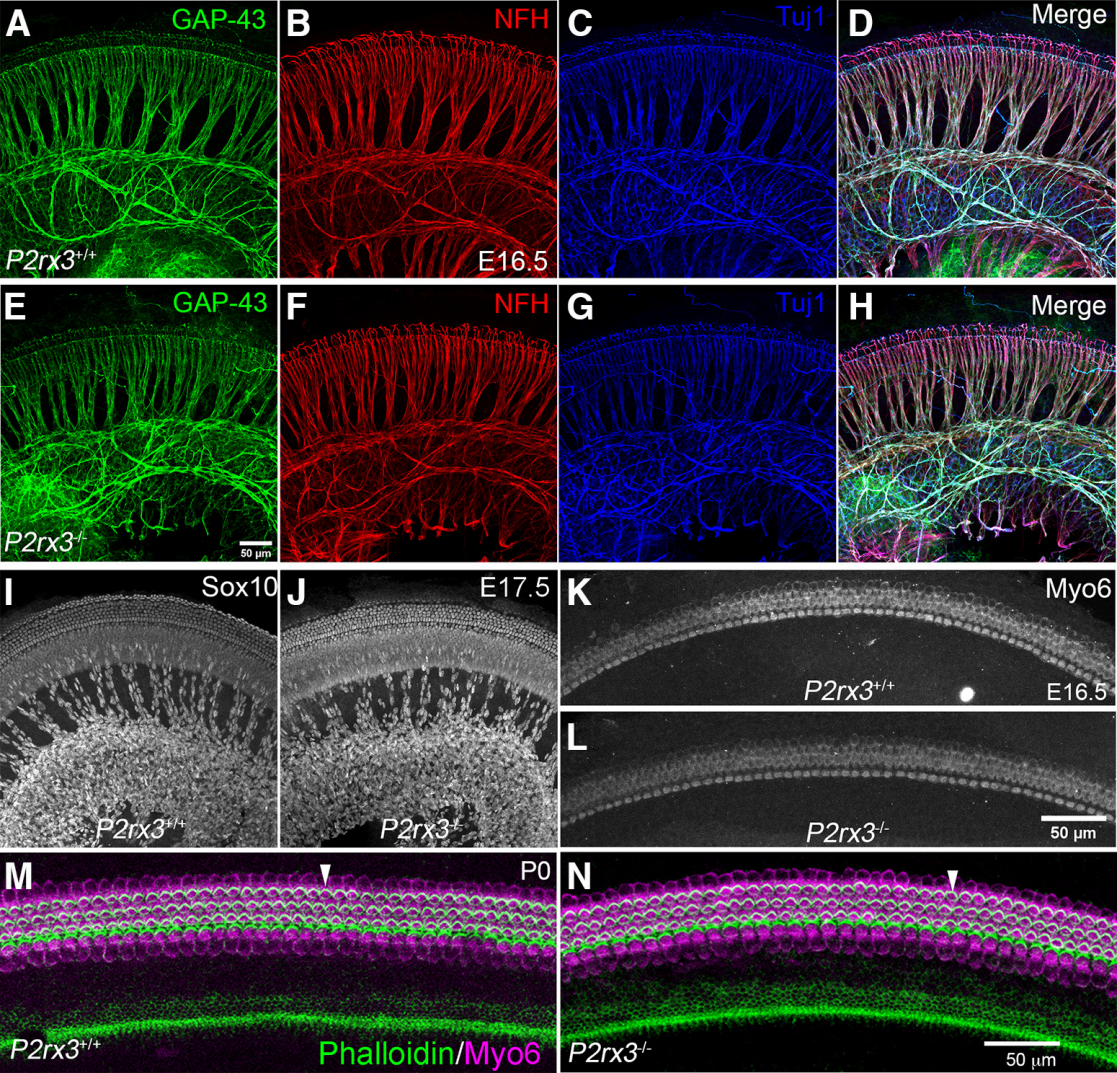
*P2rx3*−/− cochleae develop normal gross morphology. ***A–H***, WT and *P2rx3*−/− littermate cochleae at E16.5 were labeled with GAP-43 (green) to show efferent fibers, and NFH (red), and Tuj1 (blue) to show all neuronal processes. No obvious gross innervation defects were observed. ***I***, ***J***, Schwann cells labeled with Sox10 (gray) show normal distribution patterns between WT and *P2rx3*−/− cochleae at E17.5. ***K***, ***L***, Myo6 immunostaining (gray) shows no morphologic defects between littermate control and *P2rx3*−/− hair cells at E16.5. ***M***, ***N***, Stereocilia bundles labeled with Phalloidin (green) and HCs labeled with Myo6 (magenta) show no morphologic defects between WT and *P2rx3*−/− cochleae at P0.

Based on the expression of P2rx3 on SGNs and hair cells during embryonic development, we hypothesized a potential role in promoting early aspects of cochlear afferent innervation, like SGN outgrowth or refinement. Results from previous studies using cultured SGNs (Greenwood et al., 2007) and neural tube explants (Cheung et al., 2005) suggested P2rx3 may regulate axon extension. To examine this, we performed NFH immunostaining on *P2rx3*−/− cochleae and WT littermate controls at multiple embryonic stages, then measured the lengths of radial bundles, which are fasciculated SGN peripheral axons (Fig. 3). At E15.5, no significant differences were measured between the two genotypes at either apex or base in terms of peripheral axon length (Fig. 3*A–C*). Similarly, at E17.5 and P0, no differences were measurable (Fig. 3*D–I*), suggesting that *P2rx3* loss does not impair SGN peripheral axon growth. In addition, we found that treating cultured embryonic SGNs with α, β, meATP, a known P2rx3 agonist (North, 2002), led to no changes in neurite length (data not shown). These data do not support a hypothesis whereby P2rx3 affects SGN extension. The *P2rx3*−/− cochleae also lacked other general developmental defects: at all stages, cochlear length was comparable between *P2rx3*−/− cochleae and controls (data not shown). We also found that the distribution of fibers positive for GAP-43, an efferent marker (Simmons et al., 1996), was unchanged (Fig. 4*A–H*). The distribution of Sox10-positive Schwann and supporting cells also did not appear to be affected by *P2rx3* loss (Fig. 4*I*,*J*). The lack of phenotype in efferents or Schwann cells was expected given that neither of these cell types express P2rx3. Given that P2rx3 is expressed by hair cells during development, we asked whether *P2rx3*−/− cochleae showed changes in hair cell differentiation and/or patterning. As shown in Figure 4*K*,*L*, *P2rx3*−/− cochleae at E16.5 show a normal distribution of Myo6-positive hair cells. *P2rx3*−/− cochleae also show normal hair cell stereocilia bundles at P0 indicated by Phalloidin staining (Fig. 4*M*,*N*, stereocilia are noted with white arrowheads). Patterns of stereocilia were also unchanged in *P2rx3*−/− cochleae at P6 (data not shown). Overall, the loss of *P2rx3* did not alter the gross morphology of either SGNs or hair cells during the developmental period when P2rx3 is highly expressed.

### P2rx3 is necessary for normal SGNs branching patterns

**Figure 5.**
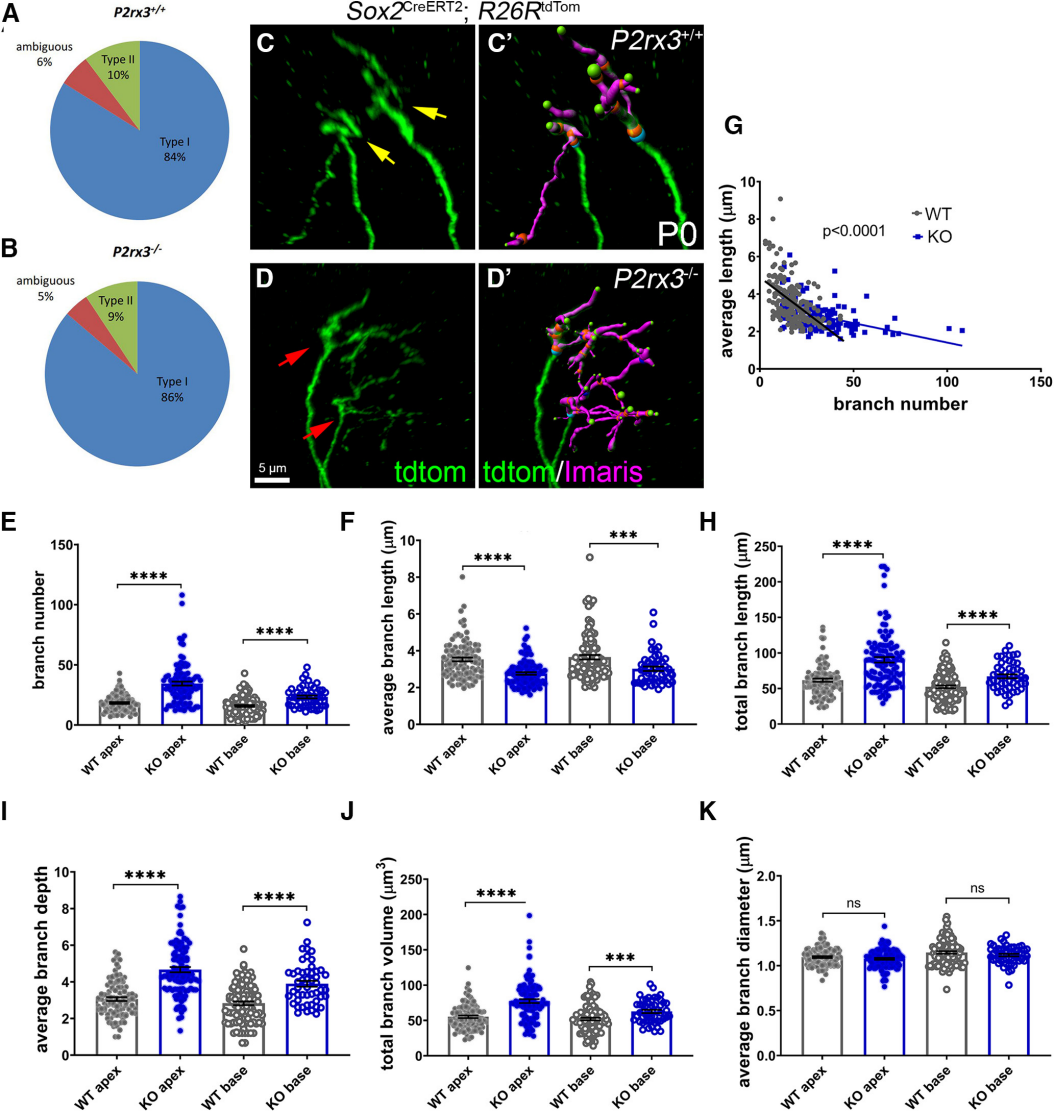
*P2rx3*−/− cochleae at P0 show more complex branching patterns at axonal terminals. ***A***, ***B***, Pie charts illustrating the composition of Type I and Type II SGNs among all sparsely labeled SGN from WT and *P2rx3*−/− cochleae at P0. ***C***, ***C’***, Representative image of Type I SGN terminal arborization from a WT cochlea at P0. Note there are two axonal terminals shown (yellow arrows). ***D***, ***D’***, Representative image of Type I SGN terminal arborization from a *P2rx3*−/− cochlea at P0. Note there are two axonal terminals shown (red arrows). ***E***, Quantification of branch number at P0. All of the following quantifications are at P0. Each datapoint represents one SGN peripheral axon (WT apex, *n* = 98, KO apex, *n* = 107, WT base, *n* = 105, KO base, *n* = 50; *N* = 8 from four WT animals, three litters and *N* = 6 from three KO animals, two litters. WT apex: 18.4 ± 0.7, KO apex: 34.5 ± 1.6, WT base: 15.8 ± 0.8, KO base: 23.4 ± 1.2; comparisons between WT and KO apex, *t*_(144)_ = 8.97, *p* < 0.0001; between WT and KO base: *t*_(90)_ = 5.37, *p* < 0.0001). ***F***, Quantification of average branch length (in μm, WT apex: 3.5 ± 0.1, KO apex: 2.8 ± 0.07, WT base: 3.7 ± 0.1, KO base: 3.0 ± 0.1; comparisons between WT and KO apex, *t*_(168)_ = 6.12, *p* < 0.0001; between WT and KO base: *t*_(135)_ = 3.70, *p* = 0.0003). ***G***, Regression plot between average branch length and branch number (WT: Y = −0.08*X + 4.9, KO: Y = −0.02*Y + 3.5). ***H***, Quantification of total branch length (in μm, WT apex: 61.7 ± 2.3, KO apex: 90.3 ± 3.8, WT base: 52.4 ± 2.0, KO base: 67.2 ± 2.8; comparisons between WT and KO apex, *t*_(172)_ = 6.39, *p* < 0.0001, between WT and KO base, *t*_(101)_ = 4.29, *p* < 0.0001). ***I***, Quantification of average branch depth (WT apex: 3.1 ± 0.1, KO apex: 4.7 ± 0.1, WT base: 2.8 ± 0.1, KO base: 3.9 ± 0.2; comparisons between WT and KO apex, *t*_(188)_ = 9.17, *p* < 0.0001, between WT and KO base, *t*_(90)_ = 5.61, *p* < 0.0001). ***J***, Quantification of total branch volume (in μm^3^, WT apex: 55.1 ± 1.8, KO apex: 77.4 ± 2.8, WT base: 51.9 ± 1.9, KO base: 62.7 ± 2.3; comparisons between WT and KO apex, *t*_(181)_ = 6.75, *p* < 0.0001, between WT and KO base, *t*_(112)_ = 3.63, *p* = 0.0004). ***K***, Quantification of average branch diameter (in μm, WT apex: 1.1 ± 0.01, KO apex: 1.1 ± 0.01, WT base: 1.1 ± 0.01, KO base: 1.1 ± 0.01; comparisons between WT and KO apex, *t*_(203)_ = 1.33, *p* = 0.1837, between WT and KO base, *t*_(129)_ = 1.47, *p* = 0.1454). n.s., not significant. ***p < 0.001; ****p < 0.0001.

Given how P2rx3 expression by SGNs during development coincides with their phase of branch refinement, we next asked whether SGNs from *P2rx3*−/− cochleae show changes in branch morphology. To do this, we bred the *P2rx3*−/− line with mice carrying *Sox2*^CreERT2^ and *R26R*^tdTomato^ and analyzed hundreds of individually-labeled SGNs. Among the tdTomato-labeled SGNs from both *P2rx3*−/− and littermate control mice, 9–10% were clearly Type II SGNs and 90–91% were clearly Type I SGNs (Fig. 5*A*,*B*). Since this is the expected distribution (Spoendlin, 1969), we believe the labeling to be stochastic. We also note that, among the labeled SGNs, a small proportion failed to extend to the sensory epithelium at P0. Since we were unable to identify these SGNs as Type I or Type II (Fig. 5*A*,*B*, “ambiguous”), they were excluded from this analysis. After reconstructing and analyzing hundreds of labeled fibers in both genotypes, we found that loss of *P2rx3* renders Type I SGNs with more complex branching patterns at P0 (Fig. 5*C*,*D*). Figure 5*C’*,*D’* shows examples of labeled SGNs with volumetric reconstructions; magenta labels indicate branch volumes; green spheres indicate branch tips. Compared with controls, SGNs from *P2rx3*−/− mice showed increased branch numbers at both the cochlear apex and base (Fig. 5*E*), suggesting P2rx3 normally controls the arborization patterns of the SGN peripheral endings. *P2rx3*−/− SGNs showed decreased average branch length values (Fig. 5*F*), which led us to plot branch number against average branch length for each SGN analyzed. After doing this, we found that terminals with large branch numbers typically have shorter individual branches, and that *P2rx3* loss exaggerates this effect (control slope = −0.08; *P2rx3*−/− slope = −0.02; Fig. 5*G*). These data suggest there is likely a redistribution of cytoskeletal structures in *P2rx3*−/− SGNs. *P2rx3*−/− SGNs also showed an increase in values for total branch length (the sum of all branch lengths; Fig. 5*H*), average branch depth (a measurement of branch complexity; Fig. 5*I*), and total branch volume (the sum of all branch volumes; Fig. 5*J*). Curiously, though, loss of *P2rx3*−/− did not appear to affect branch diameter (Fig. 5*K*). Overall, these data suggest P2rx3 normally maintains the proper size of the SGN terminal arbor at the time when hair cells become innervated.

**Figure 6.**
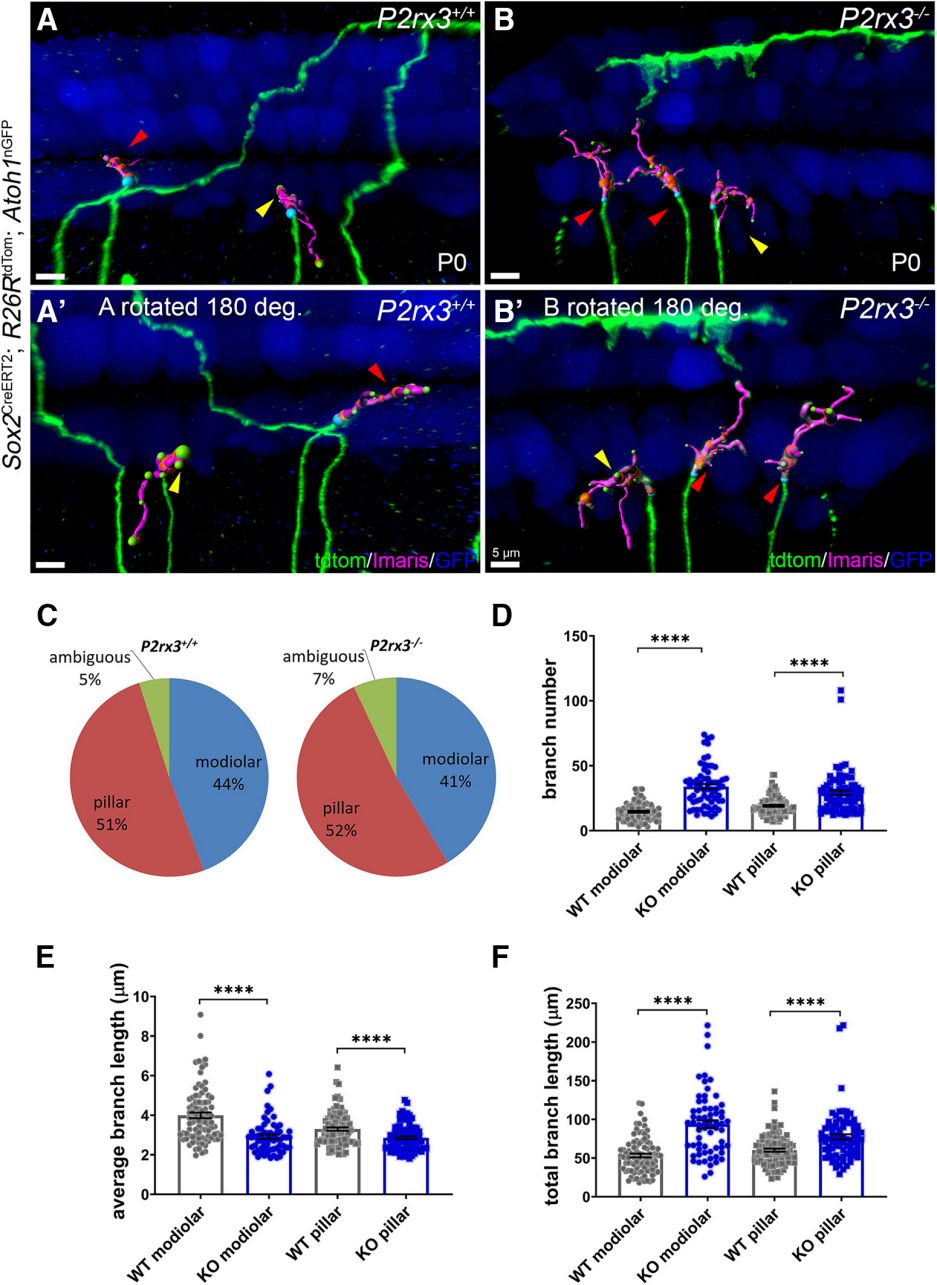
All *P2rx3*−/− SGNs show branching phenotype. ***A***, ***A’***, ***B***, ***B’***, Representative images of WT and *P2rx3*−/− Type I SGN terminals innervating the modiolar side of the inner hair cells (away from outer hair cells, yellow arrowhead) and pillar cell side of the inner hair cells (close to outer hair cells, red arrowhead) at P0, respectively. The hair cells (and some supporting cells) shown in blue are a result of *Atoh1*^nGFP^, which was carried on this line. ***C***, Pie charts illustrating the compositions of Type I SGNs among all sparsely labeled neurons from WT and *P2rx3*−/− cochleae at P0. ***D***, Quantification of branch number at P0 and all the following quantifications are at P0. Each datapoint represents one SGN terminal arborization (WT modiolar: *n* = 90, KO modiolar: *n* = 65, WT pillar: *n* = 103, KO pillar: *n* = 81; *N* = 8 from four WT animals, three litters and *N* = 6 from three KO animals, two litters. WT modiolar: 14.6 ± 0.7, KO modiolar: 33.9 ± 2.0, WT pillar: 19.2 ± 0.7, KO pillar: 29.1 ± 1.8; comparisons between WT and KO modiolar, *t*_(82)_ = 9.25, *p* < 0.0001, between WT and KO pillar, *t*_(107)_ = 5.18, *p* < 0.0001). ***E***, Quantification of average branch length (in μm, WT modiolar: 4.0 ± 0.1, KO modiolar: 2.9 ± 0.1, WT pillar: 3.3 ± 0.08, KO pillar: 2.9 ± 0.07; comparisons between WT and KO modiolar, *t*_(150)_ = 6.00, *p* < 0.0001, between WT and KO pillar, *t*_(182)_ = 3.99, *p* < 0.0001). ***F***, Quantification of total branch length (in μm, WT modiolar: 53.3 ± 2.4, KO modiolar: 93.8 ± 5.1, WT pillar: 60.2 ± 2.0, KO pillar: 77.3 ± 3.5; comparisons between WT and KO modiolar, *t*_(93)_ = 7.21, *p* < 0.0001, between WT and KO pillar, *t*_(130)_ = 4.28, *p* < 0.0001). ****p < 0.0001.

Although Type I SGNs are all similar in appearance, they are quite heterogeneous in terms of their firing characteristics and synaptic positions on inner hair cells (Liberman, 1978, 1982; Frank et al., 2009; Meyer et al., 2009; Wu et al., 2016). In particular, Type I SGNs with high rates of spontaneous discharge tend to contact the side of the inner hair cell nearest the pillar cell, whereas those with low rates of discharge tend to contact the side of the inner hair cell closest to the modiolus. Despite their elaborate branching patterns, we were easily able to distinguish whether individually-labeled Type I SGNs at P0 were positioned on either the modiolar side (Fig. 6*A*,*B*, yellow arrowheads) or pillar cell side of the inner hair cells (Fig. 6*A*,*B*, red arrowheads). This analysis was assisted by the use of *Atoh1*^nGFP^ (Lumpkin et al., 2003), which allowed us to distinguish different sides of the inner hair cell. Interestingly, the majority of SGN peripheral axons and their small branches appeared to be restricted to either the modiolar or the pillar side (and not somewhere in between) by P0. This implies that early guidance events may control the innervation location of subgroups of SGN peripheral axons as a prelude to their final differentiation. This allowed us to ask whether the loss of *P2rx3* had a more significant impact on either one of these populations. Figure 6*A’*,*B’* shows views from Figure 6*A*,*B* rotated 180° along their vertical axes. In this view, the modiolar and pillar terminations are more easily visualized. Overall, the loss of *P2rx3* did not change the proportion of Type I SGNs that contacted either side of the inner hair cell at P0 (Fig. 6*C*). Type I SGNs that terminated on the modiolar and pillar cell sides of the inner hair cell appeared to all be equally affected by the loss of *P2rx3*: both populations in *P2rx3*−/− cochleae showed increased branch numbers, decreased average length, and increased total branch length (Fig. 6*D–F*). So, at P0, while P2rx3 does not appear to control Type I SGN synaptic position, it appears to regulate branching in a way that is not specific to Type I SGNs that terminate on either side of the inner hair cell.

**Figure 7.**
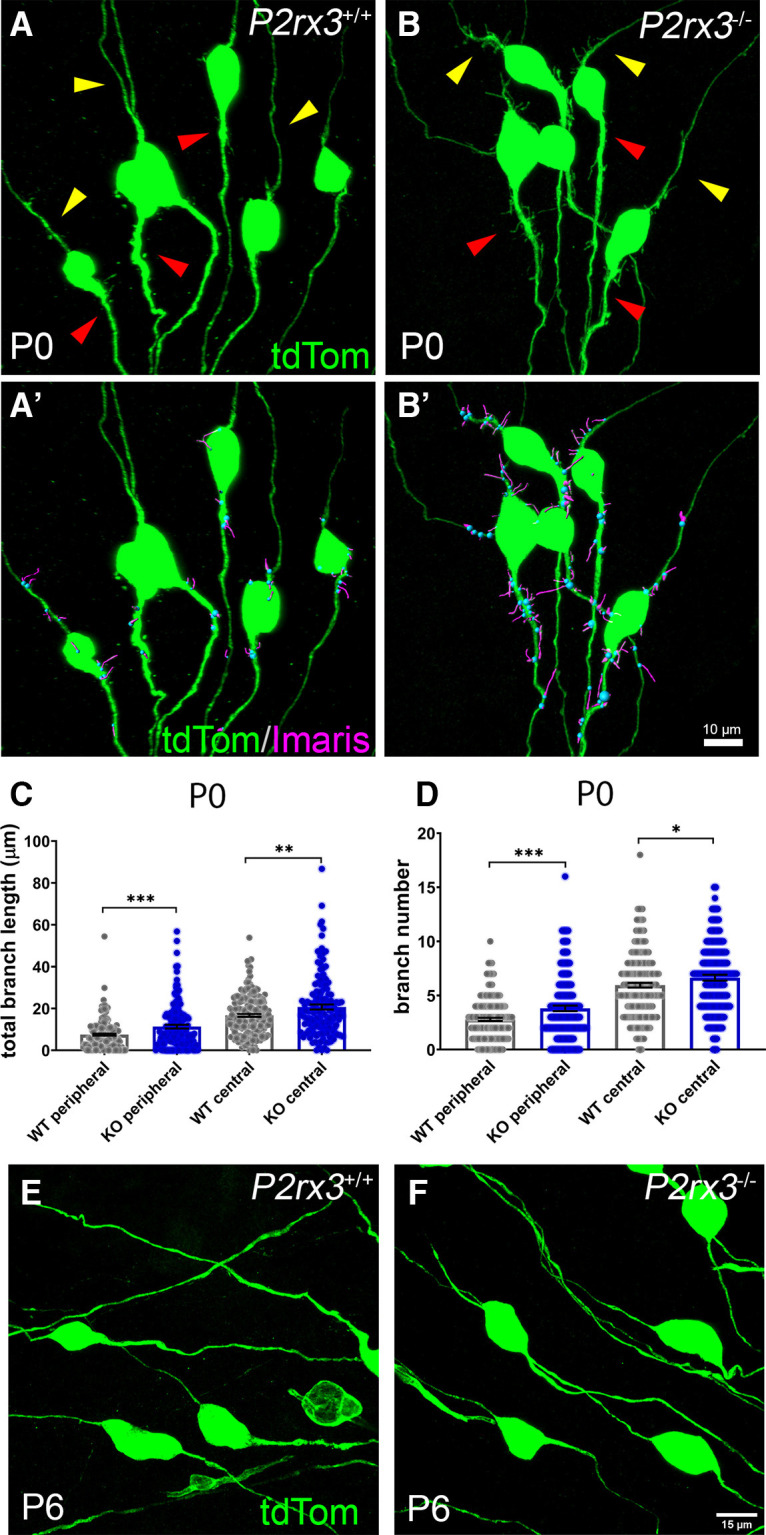
*P2rx3*−/− cochleae have more collateral branches near SGN cell bodies at P0 but not at P6. ***A***, ***A’***, ***B***, ***B’***, Representative images of *P2rx3+/+* and *P2rx3*−/− SGN cell body regions showing collateral branches on the peripheral axons (yellow arrowheads) and central axons (red arrowheads) at P0, respectively. The fluorescence signal on the cell bodies was saturated to visualize the collaterals. ***C***, Quantification of total branch length at P0 (in μm, WT peripheral, *n* = 190, KO peripheral, *n* = 148, WT central, *n* = 192, KO central, *n* = 147; *N* = 8 from four WT animals, three litters and *N* = 6 from three KO animals, two litters. WT peripheral: 7.5 ± 0.5, KO peripheral: 11.3 ± 0.9, WT central: 16.7 ± 0.7, KO central: 20.7 ± 1.2; comparisons between WT and KO peripheral, *t*_(233)_ = 3.81, *p* = 0.0002, between WT and KO central, *t*_(238)_ = 2.89, *p* = 0.0042). ***D***, Quantification of branch number at P0 (WT peripheral: 2.8 ± 0.1, KO peripheral: 3.8 ± 0.3, WT central: 6.0 ± 0.2, KO central: 6.6 ± 0.3; comparisons between WT and KO peripheral, *t*_(239)_ = 3.47, *p* = 0.0006, between WT and KO central, *t*_(303)_ = 1.99, *p* = 0.0473). ***E***, ***F***, Representative images of *P2rx3+/+* and *P2rx3*−/− SGN cell body regions showing smooth peripheral axons and central axons, respectively, at P6. *p < 0.05; **p < 0.01; ***p < 0.001.

During our analysis of the branch refinement defects at the SGN terminals in *P2rx3*−/− cochleae, we noticed that the axonal segments proximal to their cell bodies showed many small protrusions at P0 and that these protrusions (which we term “collateral branches”) seemed more numerous than what is normally seen in controls (Fig. 7). In controls, some collateral branches are localized in the main shaft of both peripheral axons and central axons while others originate from the cell bodies (Fig. 7*A*,*A’*). *P2rx3*−/− SGNs clearly showed more collateral branches, akin to the increased branch numbers seen at their peripheral terminals (Fig. 7*B*,*B’*). When we analyzed these samples, we defined the segment of the SGN axons adjacent to the SGN cell bodies, but oriented toward the hair cells as the “peripheral” segment (Fig. 7*A*,*B*, yellow arrowheads), and the segment of SGN axons adjacent to the cell bodies, but oriented toward the brainstem as the “central” segment (Fig. 7*A*,*B*, red arrowhead). Compared with controls, *P2rx3*−/− SGNs showed a significant increase of total branch length and branch number for peripheral and central segments adjacent to the cell bodies (Fig. 7*C*,*D*). To determine whether the excessive collateral branches adjacent to the cell bodies of the *P2rx3*−/− SGNs persist into later stages, we collected similar samples at P6, when P2rx3 is no longer expressed; 333 *P2rx3*−/− SGNs from seven cochleae were scored, and only 11 SGNs exhibited a few small excess collateral branches (Fig. 7*E*,*F*). Overall, the loss of *P2rx3*−/− leads to excessive collateral branches on the axonal segments around the cell bodies at P0, but these effects do not appear to be permanent.

### *P2rx3* mutants do not show persistent SGN branching defects or altered ribbon synapse distribution

**Figure 8.**
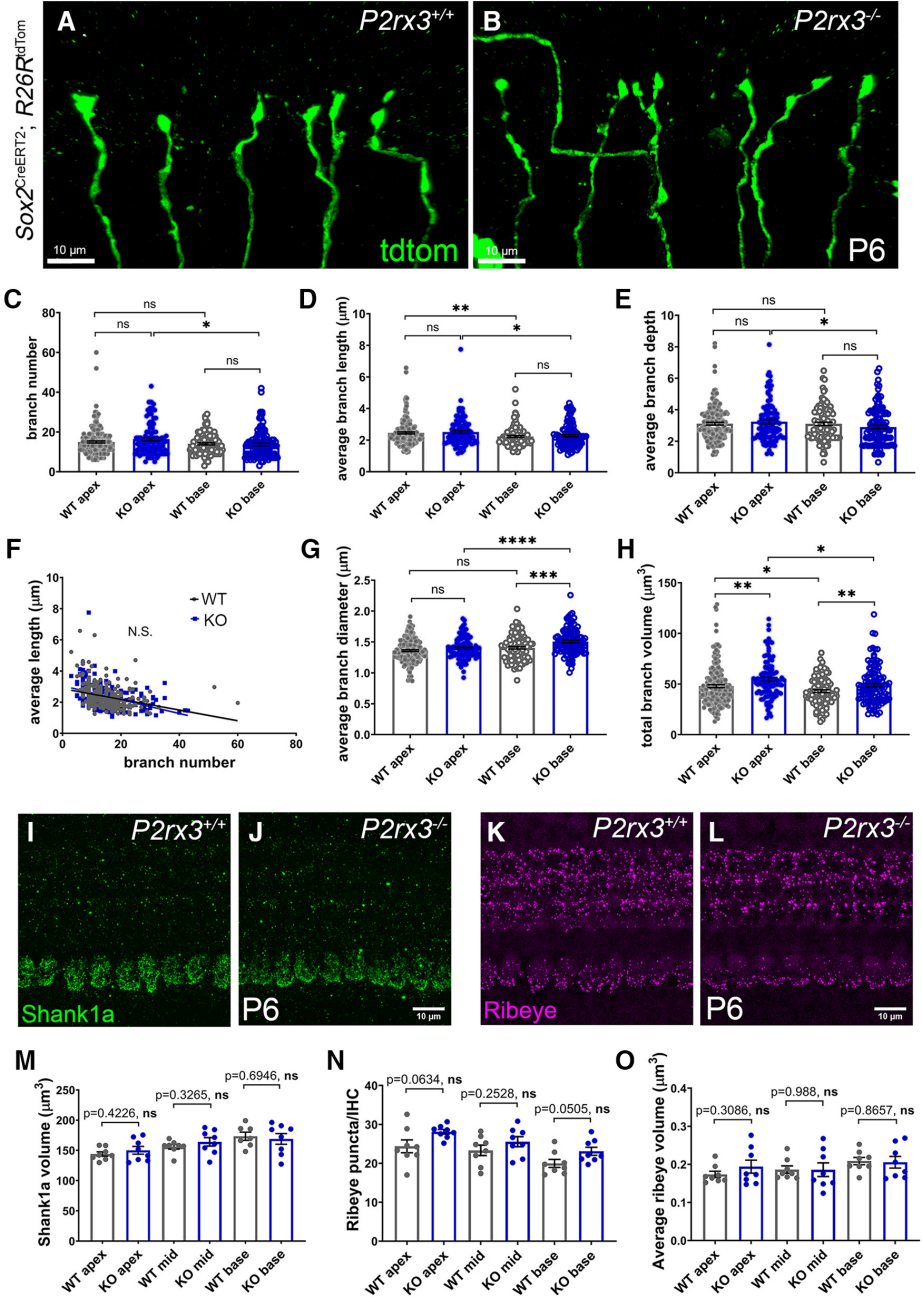
*P2rx3*−/− SGN axonal terminal branching phenotype is largely normal at P6, and *P2rx3*−/− cochleae show normal presynaptic Ribeye and postsynaptic Shank1a structures. ***A***, ***B***, Representative images of WT and *P2rx3*−/− Type I SGN terminals at P6. ***C***, Quantification of branch number at P6 and all of the following quantifications are at P6. Each datapoint represents one SGN terminal arborization (WT apex, *n* = 176, *N* = 8, KO apex, *n* = 99, *N* = 7, WT base, *n* = 106, *N* = 8, KO base, *n* = 113, *N* = 7; four WT animals from two litters and four KO animals from three litters; WT apex: 15.0 ± 0.5, KO apex: 16.4 ± 0.8, WT base: 14 ± 0.5, KO base: 13.7 ± 0.7; comparisons between WT and KO apex, *t*_(181)_ = 1.48, *p* = 0.1408, between WT and KO base, *t*_(206)_ = 0.39, *p* = 0.6991, between WT apex and base, *t*_(268)_ = 1.31, *p* = 0.19, between KO apex and base, *t*_(196)_ = 2.59, *p* = 0.0102). ***D***, Quantification of average branch length (in μm, WT apex: 2.5 ± 0.06, KO apex: 2.5 ± 0.09, WT base: 2.2 ± 0.06, KO base: 2.3 ± 0.07; comparisons between WT and KO apex, *t*_(185)_ = 0.48, *p* = 0.6351, between WT and KO base, *t*_(215)_ = 0.56, *p* = 0.5756, between WT apex and base, *t*_(263)_ = 2.77, *p* = 0.0059, between KO apex and base, *t*_(188)_ = 2.1, *p* = 0.0371). ***E***, Quantification of average branch depth (WT apex: 3.1 ± 0.08, KO apex: 3.2 ± 0.1, WT base: 3.1 ± 0.1, KO base: 2.9 ± 0.1; comparisons between WT and KO apex, *t*_(180)_ = 0.84, *p* = 0.4047, between WT and KO base, *t*_(217)_ = 1.32, *p* = 0.1899, between WT apex and base, *t*_(225)_ = 0.07, *p* = 0.9439, between KO apex and base, *t*_(202)_ = 1.99, *p* = 0.0479). ***F***, Regression plot of average branch length and branch number (WT: Y = −0.03*X + 2.9, KO: Y = −0.04*X + 3.0). ***G***, Quantification of average branch diameter (in μm, WT apex: 1.4 ± 0.01, KO apex: 1.4 ± 0.02, WT base: 1.4 ± 0.02, KO base: 1.5 ± 0.02; comparisons between WT and KO apex: *t*_(218)_ = 1.77, *p* = 0.0774, between WT and KO base, *t*_(217)_ = 3.59, *p* = 0.0004, between WT apex and base, *t*_(208)_ = 1.84, *p* = 0.0674, between KO apex and base, *t*_(210)_ = 3.99, *p* < 0.0001). ***H***, Quantification of total branch volume (in μm^3^, WT apex: 47.9 ± 1.5, KO apex: 54.6 ± 2.0, WT base: 42.9 ± 1.4, KO base: 48.7 ± 1.7; comparisons between WT and KO apex, *t*_(209)_ = 2.68, *p* = 0.0079, between WT and KO base, *t*_(209)_ = 2.64, *p* = 0.0089, between WT apex and base, *t*_(274)_ = 2.44, *p* = 0.0152, between KO apex and base, *t*_(202)_ = 2.26, *p* = 0.025). ***I***, ***J***, WT and *P2rx3*−/− cochleae at P6 were stained with Shank1a (green) to show postsynaptic scaffold structures of ribbon synapse. ***K***, ***L***, WT and *P2rx3*−/− cochleae at P6 were stained with Ribeye (magenta) to show presynaptic ribbon structures. ***M***, Shank1a volume (in μm^3^, WT apex: 144 ± 3.6, *N* = 8; KO apex: 150.1 ± 6.4, *N* = 8; WT mid: 156 ± 3.8, *N* = 8; KO mid: 164.2 ± 7.0, *N* = 8; WT base: 173.4 ± 6.7, *N* = 7; KO base: 169 ± 8.9, *N* = 8; comparison in apex: *t*_(11)_ = 0.83, in mid: *t*_(11)_ = 1.03, in base: *t*_(13)_ = 0.40, see *p* values in the figure; from eight animals, three litters, in each genotype group). Comparisons between two genotypes at apex, mid, base indicate a normal postsynaptic morphology. ***N***, ***O***, Number of Ribeye puncta per inner hair cell (WT apex: 24.4 ± 1.7, *N* = 8; KO apex: 28.1 ± 0.6, *N* = 8; WT mid: 23.3 ± 1.3, *N* = 8; KO mid: 25.6 ± 1.3, *N* = 8; WT base: 20.0 ± 1.0, *N* = 8; KO base: 23.1 ± 1.0, *N* = 8; comparison in apex: *t*_(9)_ = 2.12, in mid: *t*_(14)_ = 1.19, in base: *t*_(14)_ = 2.14, see *p* values in the figure; from eight animals, three litters, in each genotype group). Average Ribeye volume (in μm^3^, WT apex: 0.17 ± 0.008, KO apex: 0.19 ± 0.017, WT mid: 0.19 ± 0.01, KO mid: 0.19 ± 0.018, WT base: 0.21 ± 0.01, KO base: 0.21 ± 0.015; comparison in apex: *t*_(10)_ = 1.07, in mid: *t*_(11)_ = 0.015, in base: *t*_(12)_ = 0.17). Comparisons between two genotypes at apex, mid, base indicate a normal presynaptic morphology. n.s., not significant. *p < 0.05; **p < 0.01; ***p < 0.001; ****p < 0.0001.

To ask whether the SGN terminal branching defects in the *P2rx3*−/− SGNs (observed at P0) persisted through the normal phase of branching refinement (starting after P4; Fig. 1), we examined individually-labeled Type I SGNs from *P2rx3*−/− SGNs and controls from mice at P6. Like WT SGNs, *P2rx3*−/− SGNs at P6 showed very little branching and had a smooth, unramified appearance (Fig. 8*A*,*B*). After we measured their morphologic attributes (as in Fig. 5), we found that the *P2rx3*−/− SGNs showed no differences in average branch number, average branch length, and average branch depth (Fig. 8*C–E*). These data suggest that the branching phenotype in the *P2rx3*−/− SGNs largely recovers after the first postnatal week. Moreover, after plotting average branch length against branch number, we also observed no difference regarding the distribution of the data points in both genotypes (Fig. 8*F*). However, *P2rx3*−/− SGNs showed a significantly increased average branch diameter in the base and overall increased total branch volume (Fig. 8*G*,*H*), indicating some persisting effects from the loss of P2rx3 signaling. We also found some instances where morphologic measurements differed between the apex and the base in *P2rx3*−/− SGNs, whereas no apex-base differences were seen within WT SGNs. For example, for branch number and average branch depth, values in the *P2rx3*−/− SGNs at the base are consistently reduced compared with the apex (Fig. 8*C*,*E*), whereas for average branch diameter, *P2rx3*−/− SGNs showed an increase in the base (Fig. 8*G*); differences like this were not apparent in controls. So, overall, *P2rx3*−/− SGNs at P6 appear to have recovered from most of the branching defects observed at P0 but retain some modest defects. Given that P2rx3 expression stops shortly after birth (Fig. 2), there are likely compensatory mechanisms that occur toward the end of first postnatal week that also promote SGN branch refinement.

Next, we wanted to investigate whether the branch pruning defects in *P2rx3*−/− SGNs lead to abnormal ribbon synapse formation. Cochlear ribbon synapses undergo a long and dynamic maturation process starting from late embryonic stage through P30 (Yu and Goodrich, 2014; Michanski et al., 2019). To do this, we stained P6 cochleae with antibodies that mark the presynaptic factor Ribeye, and antibodies that mark the postsynaptic factor Shank1a (Huang et al., 2012). In terms of the postsynaptic environment, Shank1a positive scaffold structures appear cup-shaped at the bottom of the inner hair cells at P6 (Fig. 8*I*,*J*). Compared with controls, we found no differences in *P2rx3*−/− cochlea regarding Shank1a volume (Fig. 8*M*), surface area and sphericity (data not shown). These data suggest that postsynaptic structures in the *P2rx3*−/− cochlea at P6 are normal. In terms of the presynaptic environment, numerous ribbon bodies localize to the bottom of the inner hair cells with a few dispersed throughout the cytoplasm (Yu et al., 2013). Overall, *P2rx3*−/− cochleae showed similar Ribeye number and average Ribeye volume compared with WT cochlea (Fig. 8*N*,*O*). Therefore, despite the possibility that excessive branches and terminal endings might cause malformed synaptic connections between SGN peripheral axons and inner hair cells, we report that *P2rx3*−/− cochleae exhibited a normal distribution of presynaptic and postsynaptic ribbon synapse structures. This is consistent with the observed recovery of SGN terminal branches in *P2rx3*−/− cochleae at P6.

### *P2rx3*−/− cochleae show altered proportions of Type I SGN subtype markers

**Figure 9.**
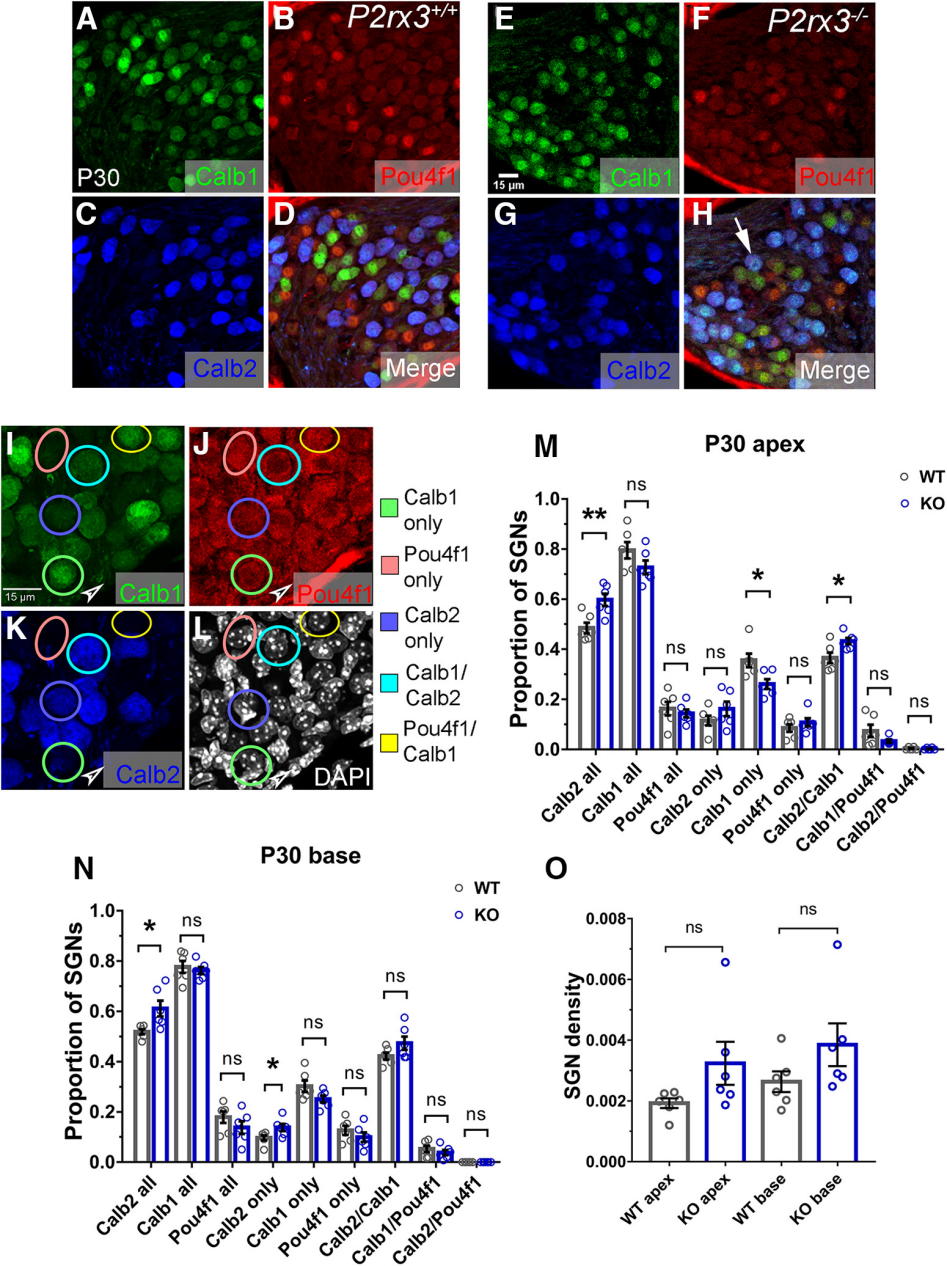
*P2rx3*−/− cochleae show altered proportions of Type I SGN subtype markers. ***A–H***, Representative images of WT and *P2rx3*−/− cochlear cross-sections at P30 labeled with Calb1, Pou4f1, and Calb2 to show three Type I SGN subtypes, respectively. White arrow indicates a Calb1/Calb2-double positive neuron. ***I–L***, Representative images of a P30 cochlear cross-section immunolabeled with Calb1, Pou4f1, Calb2, and DAPI to illustrate quantification of the three Type I SGN subtypes. Circled SGNs are the same for each image. Circles indicate examples of cells counted as Calb1-positive, Pou4f1-positive, Calb2-positive, Calb1/Calb2-double positive, or Pou4f1/Calb1-double positive. Arrowheads indicate stained areas lacking SGNs; cells like these were used to monitor background fluorescence levels. ***M***, Comparisons of subtype proportion in each group. Each 95% CI indicates the difference in means between WT and *P2rx3*−/− in each group. *P2rx3+/+* and *P2rx3*−/− cochlear apex (WT Calb2 all: 0.485 ± 0.021, KO Calb2 all: 0.597 ± 0.025, *t*_(10)_ = 3.46, 95% CI [0.03984, 0.1854], *p* = 0.0063; WT Calb1 all: 0.795 ± 0.033, KO Calb1 all: 0.727 ± 0.027, *t*_(10)_ = 1.6, 95% CI [−0.1638, 0.02706], *p* = 0.141; WT Pou4f1 all: 0.163 ± 0.027, KO Pou4f1 all: 0.144 ± 0.016, *t*_(8)_ = 0.62, 95% CI [−0.09136, 0.05251], *p* = 0.5512; WT Calb2 only: 0.116 ± 0.019, KO Calb2 only: 0.162 ± 0.029, *t*_(9)_ = 1.34, 95% CI [−0.03249, 0.1255], *p* = 0.2147; WT Calb1 only: 0.356 ± 0.027, KO Calb1 only: 0.261 ± 0.02, *t*_(9)_ = 2.83, 95% CI [−0.1694, −0.01909], *p* = 0.0194; WT Pou4f1 only: 0.086 ± 0.015, KO Pou4f1 only: 0.108 ± 0.016, *t*_(10)_ = 1.02, 95% CI [−0.02699, 0.07275], *p* = 0.3306; WT Calb2/Calb1: 0.365 ± 0.021, KO Calb2/Calb1: 0.433 ± 0.013, *t*_(8)_ = 2.72, 95% CI [0.01045, 0.1239], *p* = 0.0257; WT Calb1/Pou4f1: 0.074 ± 0.024, KO Calb1/Pou4f1: 0.033 ± 0.006, *t*_(6)_ = 1.64, 95% CI [−0.1039, 0.02136], *p* = 0.1559; WT Calb2/Pou4f1: 0.0036 ± 0.0023, KO Calb2/Pou4f1: 0.0026 ± 0.0016, *t*_(9)_ = 0.36, 95% CI [−0.007409, 0.005352], *p* = 0.7241;Welch’s *t* test, *N* = 6 from six WT animals, two litters and *N* = 6 from six KO animals, three litters). ***N***, Comparisons of subtype proportion in each group in WT and *P2rx3*−/− cochlear base (WT Calb2 all: 0.519 ± 0.01, KO Calb2 all: 0.611 ± 0.032, *t*_(6)_ = 2.8, 95% CI [0.01163, 0.1734], *p* = 0.0312; WT Calb1 all: 0.777 ± 0.024, KO Calb1 all: 0.763 ± 0.014, *t*_(8)_ = 0.51, 95% CI [−0.07792, 0.04968], *p* = 0.6243; WT Pou4f1 all: 0.179 ± 0.023, KO Pou4f1 all: 0.138 ± 0.025, *t*_(10)_ = 1.21, 95% CI [−0.118, 0.03495], *p* = 0.2538; WT Calb2 only: 0.097 ± 0.01, KO Calb2 only: 0.138 ± 0.014, *t*_(9)_ = 2.37, 95% CI [0.001816, 0.08083], *p* = 0.0422; WT Calb1 only: 0.302 ± 0.023, KO Calb1 only: 0.251 ± 0.014, *t*_(8)_ = 1.92, 95% CI [−0.1121, 0.01011], *p* = 0.0908; WT Pou4f1 only: 0.127 ± 0.018, KO Pou4f1 only: 0.099 ± 0.019, *t*_(10)_ = 1.04, 95% CI [−0.08538, 0.03097], *p* = 0.3219; WT Calb2/Calb1: 0.422 ± 0.014, KO Calb2/Calb1: 0.473 ± 0.026, *t*_(8)_ = 1.72, 95% CI [−0.01819, 0.1206], *p* = 0.1261; WT Calb1/Pou4f1: 0.053 ± 0.013, KO Calb1/Pou4f1: 0.038 ± 0.01, *t*_(10)_ = 0.88, 95% CI [−0.05097, 0.02231], *p* = 0.4023; WT Calb2/Pou4f1: 0, KO Calb2/Pou4f1: 0; Welch’s *t* test). ***O***, Quantification of SGN density (WT apex, 0.0019 ± 0.00016, *N* = 6; KO apex, 0.0032 ± 0.0007, *N* = 6, *t*_(6)_ = 1.81, *p* = 0.1246, 95% CI [−0.0005, 0.003]; WT base, 0.0026 ± 0.0003, *N* = 6; KO base, 0.0038 ± 0.0007, *N* = 6, *t*_(7)_ = 1.56, *p* = 0.1615, 95% CI [−0.0006, 0.003]). n.s., not significant. *p < 0.05; **p < 0.01.

Recent single-cell RNA sequencing analyses have identified three subpopulations of Type I SGNs (Petitpré et al., 2018; Shrestha et al., 2018; Sun et al., 2018). The three populations are distinguishable by various molecular markers and differentiation is dependent on activity as a result of input from inner hair cells (Shrestha et al., 2018; Sun et al., 2018). Considering that ATP signaling is critical for spontaneous activity in the developing cochlea (Tritsch et al., 2010; Wang and Bergles, 2015), and that P2rx3 appeared to control normal patterns of branching and maturation during development (Figs. 5-7 here), we predicted that loss of *P2rx3* would impair Type I SGN differentiation. To test this, we collected P30 WT and *P2rx3*−/− cochleae and stained cross-sections simultaneously with antibodies that identify Calbindin (Calb1), Calretinin (Calb2), and the transcription factor Pou4f1. These factors were shown previously to delineate the three subpopulations of Type I SGNs (Petitpré et al., 2018; Shrestha et al., 2018; Sun et al., 2018), but it was also clear that some Type I SGNs expressed more than one of these factors. While the presence of any combination of these factors does not necessarily indicate functional attributes of the SGNs, any changes in their proportions between groups could indicate altered differentiation. Here, we devised a method of staining and scoring samples to enable us to account for SGNs positive for one or multiple markers (Fig. 9*A–H*; see Materials and Methods; Brooks et al., 2020). This method is illustrated in Figure 9*I–L*, where colored circles indicate examples of SGNs counted as positive for one or more marker. The green, salmon, and dark blue circles respectively indicate SGNs positive for Calb1 only, Pou4f1 only, or Calb2 only. The light blue circle highlights an SGN double positive for Calb1 and Calb2; the SGN circled in yellow is double positive for Pou4f1 and Calb1. As shown in Figure 9*M*,*N*, we found that the proportion of SGNs expressing Calb2 (Calb2 all) was increased in the *P2rx3*−/− cochleae by 11% in the apex and 9.2% in the base. In the apex of *P2rx3*−/− cochleae, there was a corresponding decrease in SGNs positive only for Calb1, and an increase of SGNs positive for both Calb1 and Calb2 (Fig. 9*M*,*N*). The base of *P2rx3*−/− cochleae showed a different scenario: the increase in the entire population of Calb2 expressing cells manifested as a result of an increase in cells positive for Calb2 only. To examine the possibility these differences might be because of SGN death, we also compared SGN density between *P2rx3*−/− cochleae and controls and found no differences (Fig. 9*O*). Thus, loss of *P2rx3* leads to an increase in the number of Calb2-positive cells. Previously, it was shown that, when Type I SGNs differentiate, they tend to express just one of these factors (Petitpré et al., 2018; Shrestha et al., 2018; Sun et al., 2018). Given this and the increase in numbers of Calb2-positive neurons in the *P2rx3*−/− cochleae, these data support a model whereby P2rx3 may provide a modest contribution to Type I SGN differentiation.

## Discussion

**Figure 10.**
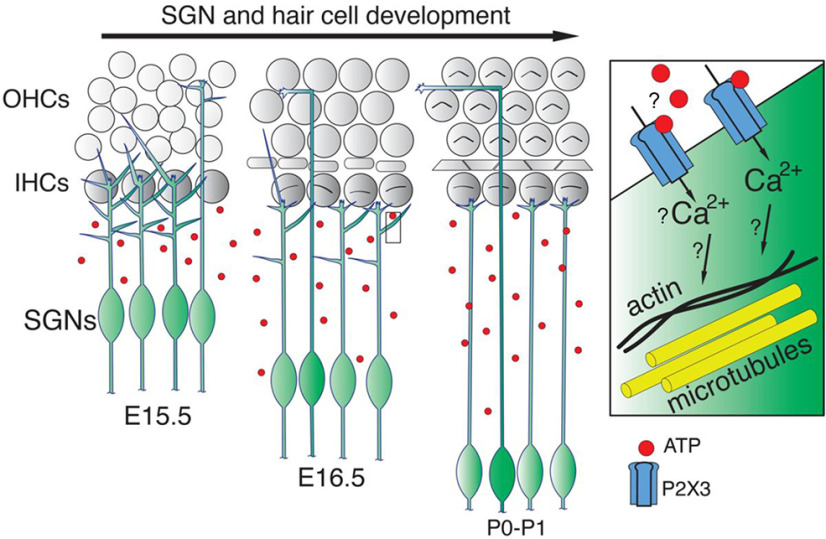
A model for the role of P2rx3 in SGN branch refinement. Cartoon schematic illustrating a model whereby P2rx3 receptors in SGNs possibly detect extracellular ATP, and possibly generate calcium signals that regulate SGN branch refinement.

SGNs are the primary afferent neurons that connect the peripheral and the central auditory systems (Appler and Goodrich, 2011; Nayagam et al., 2011; Coate and Kelley, 2013). Making precise connections between SGNs and sensory hair cells depends on numerous events, such as the correct targeting of axonal terminals to either inner or outer hair cells (Coate et al., 2015; Druckenbrod and Goodrich, 2015). One critical aspect of cochlear wiring is that each SGN peripheral axon must refine its elaborate terminal arborization to form a one-to-one connection with a single inner hair cell. Understanding these events in the auditory system will help elucidate how complex neural circuits in other parts of the nervous system refine their connectivity patterns (Jan and Jan, 2003). In this report, we have defined the developmental time course of SGN peripheral axon branching refinement and found that P2rx3 is an important player in this process (Fig. 10). Future studies will determine the extent to which the action of P2rx3 in this process is dependent on ATP, and what downstream signaling events in SGNs might be initiated by P2rx3 (Fig. 10). P2rx3 receptors are known to be expressed in a variety of afferent sensory neurons and have been shown to play an essential roles in their excitability (Housley et al., 2009). For example, in the urinary system, P2rx3 was shown to be expressed by pelvic afferent nerves and is necessary for their activity *in vitro* (Vlaskovska et al., 2001) and *in vivo* (Cockayne et al., 2000). P2rx3 is also expressed by, and necessary for, the activation of nociceptors (Souslova et al., 2000; Tsuda et al., 2000), taste afferents (Finger et al., 2005; Vandenbeuch et al., 2015), gut afferents (Bian et al., 2003), and many others. As shown here, P2rx3 is expressed by SGNs and hair cells in the cochlea but is eliminated well before the onset of hearing (Fig. 2). So, it likely does not participate in SGN excitation, which is known to be mediated mainly by AMPA signaling (Glowatzki and Fuchs, 2002), and the extent to which P2rx3 contributes to prehearing spontaneous activity in the cochlea remains to be seen. To our knowledge, a role in neuron branching refinement, as shown here (Figs. 5, 6), represents a novel function for P2rx3.

Here, we showed distinct temporal and spatial expression patterns of P2rx3 receptors in the developing cochlea. Using anti-P2rx3 antibody staining, we showed that SGNs express P2rx3 receptors on their cell bodies and neuronal processes from E12.5 to P6. This period of development corresponds to when SGNs migrate, form contacts with inner and outer hair cells, and undergo branching refinement (Fig. 1). P2rx3 expression by inner and outer hair cells is considerably more transient: inner hair cells express P2rx3 from E15.5 to E18.5, and outer hair cells express P2rx3 from E16.5 to P3. Our results align with and extend previous findings of dynamic P2rx3 receptor expression from E18 to P6 in the mouse cochlea (Huang et al., 2006). In terms of when P2rx3 is turned off, *P2rx3* mRNA is mostly undetectable in SGNs after P6 (Lu et al., 2011; Li et al., 2020), and this corresponds with the elimination of P2rx3 protein expression (Fig. 2). The reduction of P2rx3 is likely a result of *P2rx3* transcriptional downregulation but could also be related to ligand-activated endocytosis from the plasma membrane (Vacca et al., 2009). The expression of P2rx3 in hair cells appears to follow a similar time course as Atoh1, which is expressed by all hair cells transiently and early in their development (Driver et al., 2013). Given that no obvious morphologic abnormality was observed from our immunostaining studies of hair cells, it appears that *P2rx3* is dispensable for their morphogenesis. What remains to be determined is the extent to which the SGN phenotypes we observed in the *P2rx3*−/− cochleae resulted from a lack of P2rx3 in SGNs, hair cells, or both. Future investigations of cell type-specific loss of *P2rx3* will shed light on this important issue.

Overall, the data here suggest an inhibitory role for P2rx3 in maintaining the proper size of the SGN terminal arborization. Since P2X receptors are known to induce calcium signals (Khakh and North, 2012), we predict that P2rx3 activation normally leads to calcium transients that modulate the SGN cytoskeleton as it matures (Fig. 10). In these studies, the earliest developmental stage of *P2rx3*−/− cochleae we examined using the sparse labeling approach was P0. Whether *P2rx3*−/− SGNs show excessive branches earlier than P0 remains to be determined. Since P2rx3 is expressed as early as E12.5 in SGNs (Fig. 2), it could regulate aspects of branch formation in addition to refinement. In addition, some cellular mechanisms of SGN branch refinement remain unclear: does each small branch retract into the main branch, or does each small branch fragment and slough off into the extracellular space? To approach questions like this and gain further insights into how P2rx3 receptors contribute to SGN terminal morphogenesis, time-lapse imaging studies will need to be conducted. As shown in Figure 9, we also found that cochleae lacking *P2rx3* showed a modest change in how Type I SGN subtype markers were represented. In particular, *P2rx3*−/− cochleae at P30 showed increased numbers of SGNs that were positive for Calb2. Based on previous work showing that Type I SGN subtypes become distinguishable by marker expression increasingly over developmental time (Shrestha et al., 2018; Sun et al., 2018), this phenotype possibly represents reduced Type I SGN differentiation. Extensive profiling of Type I SGN markers and firing characteristics would need to be done to determine whether *P2rx3* loss leads to any functional changes. As noted below, it is also possible that the increased proportion of Calb2-positive cells in *P2rx3*−/− cochleae could have resulted from altered Ca^2+^ fluctuations.

Previously, P2rx3 was proposed to inhibit SGN axon outgrowth (Greenwood et al., 2007), but our data here suggest this is not the case: *P2rx3*−/− cochleae showed no differences in radial bundle length compared with controls. However, it is possible that redundant mechanisms might compensate for the loss of *P2rx3* in the context of axon outgrowth. Given the branch morphology phenotype in *P2rx3*−/− cochleae, it was surprising that SGNs lacking P2rx3 did not show changes in synaptic structures (Fig. 8). It is possible that our staining methods were not sensitive enough to detect subtle changes in ribbon synapse compositions or organization in *P2rx3*−/− cochleae, or it is possible that any defects recovered by P6 along with the branching defects (Fig. 8).

What are the mechanisms by which P2rx3 controls the dynamics of SGN branch refinement? One possibility is that P2rx3 signaling may interact with Sema5B/PlexinA1 downstream signals, which was shown previously to serve a role in SGN branching refinement (Jung et al., 2019). Another possibility is that SGN microtubules are normally destabilized by P2rx3-mediated increases in calcium (Liu and Dwyer, 2014). In this case, loss of P2rx3 signaling might lead to more stabilized, and thus more numerous branches. There are also numerous cytosolic and adhesion factors that respond to calcium to modulate filopodial dynamics during growth cone guidance (Gomez et al., 2001; Gomez and Zheng, 2006; Rosenberg and Spitzer, 2011). Since P2rx3 likely regulates calcium entry into the SGNs, any one of these factors might participate in SGN branch regulation downstream of P2rx3. In addition, nerve growth factor (NGF) signaling was shown to regulate P2rx3-mediated synaptic activity by decreasing the phosphorylation of threonine residues on P2rx3, which altered its assembly on the membrane (D’Arco et al., 2007). Although NGF has not been reported in the cochlea, it is possible that it or other trophic cues might modulate P2rx3 activity. Future work will need to dissect the molecular mechanisms of how P2rx3 signaling in SGNs regulates how their branches are refined during hair cell innervation.

Previously, it was shown that P2rx3 expression is regulated by several mechanisms (Giniatullin et al., 2008). For example, in trigeminal nociceptive neurons, calcitonin gene-related peptide (CGRP) upregulated *P2rx3* transcription and membrane trafficking via protein kinase A (PKA; Fabbretti et al., 2006). Another study showed that brain-derived neurotrophic factor (BDNF), calcium/calmodulin-dependent kinase II (CaMKII), and cAMP-response element-binding protein (CREB) were involved in *P2rx3* transcriptional regulation (Simonetti et al., 2008). Other *P2rx3* regulators could include C-terminal Src inhibitory kinase (Csk; D’Arco et al., 2009), cyclin-dependent kinase-5 (Cdk5; Nair et al., 2010), and calcium/calmodulin-dependent serine protein kinase (CASK; Gnanasekaran et al., 2013). In future studies, it will be important to determine whether similar or different mechanisms exist in the cochlea.

The data in this report add to an already existing body of knowledge on purinergic signaling in the developing cochlea. Like other parts of the nervous system such as retina, spinal cord, and hippocampus, which display input-independent spontaneous activity for establishing mature neural circuits (Blankenship and Feller, 2010), the developing cochlea also exhibits spontaneous activity before hearing onset (Wang and Bergles, 2015). Purinergic signaling is known to play an essential role in this aspect of cochlear development (Tritsch et al., 2010). Briefly, ATP released from inner supporting cells activates P2ry1 auto-receptors to release calcium from internal stores, which then opens TMEM16A chloride channels (Wang et al., 2015; Babola et al., 2020). Subsequent potassium release depolarizes inner hair cells, which then propagates spontaneous activity into the brain by releasing glutamate onto Type I SGNs (Babola et al., 2018). There is a large body of evidence that disrupted spontaneous activity patterns impairs refinement of sensory maps (Kandler et al., 2009; Kirkby et al., 2013). In the developing auditory brainstem, the loss of efferent cholinergic neurotransmission alters temporal patterns of spontaneous activity and disrupts topographic refinement of synaptic connections and pruning of axon terminals (Clause et al., 2014). In future work, we will determine the extent to which Ca^2+^ transients are altered in *P2rx3*−/− SGNs, which would link P2rx3 and Ca^2+^ to SGN branch refinement. Additionally, since Calb2 is a Ca^2+^ binding protein (Schwaller, 2009), changes to the rate or spatial distribution of Ca^2+^ buffering in *P2rx3*−/− SGNs might underlie the more widespread distribution of Calb2 or somehow change its program of expression.

Recently, outer hair cells were also shown to exhibit spontaneous calcium transients, which were stimulated by neighboring Deiters’ cells. Pharmacological blockade of P2rx3 receptors inhibited the firing dynamics of outer hair cells, and this altered normal patterns of outer hair cell ribbon synapse distribution and innervation (Ceriani et al., 2019). Extracellular ATP is also known to excite Type II SGNs (Weisz et al., 2009), and recent studies have shown that purinergic signaling occurs in Type II SGNs in response to hair cell damage (Liu et al., 2015). P2rx3 protein is expressed by all developing SGNs, and it will be important to determine in future studies if P2rx3 serves a similar role in the branching behavior of Type II SGNs as it does in Type Is. The scarcity of singly labeled Type II SGNs in the *Sox2*^CreERT2^; *R26R*^tdTom^ cochleae prevented us from comprehensively studying how Type II SGNs were affected by P2rx3 in this study.

Purinergic signaling has been implicated in a neuro-modulatory role beyond cochlea in the auditory system. P2X receptor expressing bushy cells in the cochlear nucleus showed ATP evoked action potentials and calcium signals. Endogenous extracellular ATP not only facilitates spontaneous activity, but also sound-evoked activity largely during developmental stages (Dietz et al., 2012). It seems that neurotransmission mediated by extracellular purines is a primitive form of neuronal communication in addition to the dominant glutamatergic transmission. Indeed, ATP modulation matures in a high-to-low frequency pattern in the cochlear nucleus, and this is mediated by P2rx2/3 receptors, which decrease in activity following development (Jovanovic et al., 2017). In this study, we did not examine the function of P2rx3 in the wiring of the SGN central axons (which do express P2rx3; Fig. 2) with neurons in the cochlear nucleus. In future studies, it will be important to determine whether SGN branching and refinement during connectivity in the cochlear nucleus, as in the periphery, is dependent on P2rx3.

Whether loss of *P2rx3* leads to hearing impairment remains an open question. Interestingly, one of the closely related family members of P2rx3, P2rx2, has been implicated in hearing loss associated with DFNA41 (Yan et al., 2013). *P2rx2* point mutants lacked ATP-evoked inward currents and ATP-stimulated membrane permeability. Both humans carrying this mutation and *P2rx2* null mice showed progressive high-frequency hearing loss (Yan et al., 2013). In a separate study, *P2rx2* null mice failed to develop temporary threshold shift (TTS) making them more susceptible to permanent hearing loss because of synaptic damage (Housley et al., 2013). P2rx2 receptors are known to form heteromeric trimers with P2rx3 (Jiang et al., 2003), but given that P2rx2 does not seem to be expressed in embryonic SGNs or HCs, it is not likely that functional P2rx2-containing receptors play a role in early SGN development. Given there is no apparent alteration in SGN numbers or hair cell synaptic contacts in mice lacking *P2rx3* (Figs. 8, 9), it is doubtful they would show any measurable auditory brainstem response (ABR) threshold shift. It is also doubtful that the modest change in SGN subtype marker distribution (Fig. 9) would lead to any ABR threshold changes. Nevertheless, it will be important in future studies to examine whether any of the complex aspects of auditory detection in the ascending auditory system are dependent on P2rx3.

## Synthesis

Reviewing Editor: Lynda Erskine, University of Aberdeen Decisions are customarily a result of the Reviewing Editor and the peer reviewers coming together and discussing their recommendations until a consensus is reached. When revisions are invited, a fact-based synthesis statement explaining their decision and outlining what is needed to prepare a revision will be listed below. The following reviewer(s) agreed to reveal their identity: NONE. Note: If this manuscript was transferred from JNeurosci and a decision was made to accept the manuscript without peer review, a brief statement to this effect will instead be what is listed below.

As you will see from the comments below, both reviewers are agreed that your work makes an important advance in the field and that the manuscript has been improved substantially. However, we are agreed that the manuscript would benefit from same additional small changes to the text to clarify the significance of some findings. The comments of both reviewers are appended below. Thank you for submitting your interesting work to eNeuro.

Reviewer 1:

As far as I am aware, a developmental role for purinergic receptors on the pathfinding and molecular phenotype of spiral ganglion neurons has not previously been studied.

The authors have adequately addressed my comments provided during the first round of review.

Reviewer 2:

The role of purinergic signaling in cochlear development holds high significance for the field, due both to the known role of ATP in spontaneous activity and the presence of purinergic receptors in developing and mature cochlear cells. This paper reports an extensive, compelling, and well-quantified analysis of the effects of losing one particular purinergic receptor, P2rx3. The data provide new insights into the fine details of how SGN processes branch developmentally and show that P2rx3 plays a role in these events.

The authors have done a nice job incorporating the reviewers’ suggestions. The quality of the wholemount stains is outstanding and the analysis they have done, using Sox2-CreER, is both appropriate and illuminating for studying afferent process pruning events in the developing cochlea. Although the authors softened the language related to the change in Type I SGN subtype markers, some issues still remain, as outlined below. To be clear, the data are fine (though some questions about the statistics need to be addressed - see below). The problem is simply on the overreliance of a few markers to say anything about SGN identity, when the effect reported could have nothing to do with identity at all and this point still needs to made more clearly. This can be dealt with easily with a few more tweaks to the text. Please note that I am pushing this point simply to make sure that readers appreciate the nuances of what this all could mean, not to criticize the data themselves (which are beautiful).

Major issues:

Figure 9 is still concerning. As indicated in the previous reviews, the authors need to consider the possibility that the phenotype is secondary. They avoid the use of the word specification appropriately, but I don’t think that the word differentiation solves the problem. This point was raised earlier, but let me try to be more explicit this time.

Here’s the data reported in Figure 9 legend (for 9I):

a. WT Calb2: 0.12 {plus minus} 0.017, KO Calb2: 0.15 {plus minus} 0.02, 95% CI [-0.093, 0.036], p = 0.7908;

b. WT Calb1: 0.37 {plus minus} 0.027, KO Calb1: 0.29 {plus minus} 0.011, 95% CI [0.015, 0.14], p = 0.0088;

c. WT Pou4f1: 0.1 {plus minus} 0.013, KO Pou4f1: 0.09 {plus minus} 0.012, 95% CI [-0.056, 0.073], p = 0.9996

d. WT Calb2/Calb1: 0.34 {plus minus} 0.023, KO Calb2/Calb1: 0.44 {plus minus} 0.028, 95% CI [-0.16, -0.035], p = 0.0006;

e. WT Calb1/Pou4f1: 0.069 {plus minus} 0.012, KO Calb1/Pou4f1: 0.033 {plus minus} 0.0078, 95% CI [-0.029, 0.1], p = 0.581;

f. WT Calb2/Pou4f1: 0.0081 {plus minus} 0.003, KO Calb2/Pou4f1: 0.0033 {plus minus} 0.0016, 95% CI [-0.06, 0.069], p > 0.9999

I calculated the following based on the data above (not counting those overlapping with Pou4f1 because they are relatively small proportions):

Total proportion of cells expressing any Calb2 (exclusive + non-exclusive): 0.12 + 0.34 = 0.46 (WT); 0.15 + 0.44 = 0.59 (KO)

Total proportion of cells expressing any Calb1: 0.37 + 0.34 = 0.71 (WT) and 0.29 + 0.44 = 0.73 (KO)

Thus, the reported change in overlapping Calb2/Calb1 expression can be attributed to ‘expansion’ of Calb2 expression: there is a 28% increase in the proportion of cells deemed to be Calb2+ (exclusive or not). There is no change in proportion of cells expressing Calb1. The difference they report in cells expressing Calb1 only (which is statistically significant) can also result simply from Calb2 proportion being expanded. Therefore, this is the primary phenotype. This is how their results should be reported.

Now, Calb2 is normally expressed in a gradient from Ia to Ic SGNs. Given this, it is critical to report the criteria for assigning cells as Calb2+ vs. Calb2-. As you might imagine, inconsistency in applying cut-offs could easily suggest a proportion change when in fact the only change is the level of Calb2. Especially given the known effect of purinergic signaling on calcium levels, it is easy to envision cells homeostatically increasing Calb2 levels, since Calb2 is a calcium binding protein. I’m not sure I would call this a sign of altered differentiation, but that may be a matter of semantics. In any case, the authors should report how they chose their cut-offs for each of their markers. Did they account for background noise differences in their images in setting thresholds? Did they account for variation in antibody penetration in tissue sections? Were they blind to genotype when they did these analyses? All of these should be stated clearly and the authors should re-analyze their data if these aspects had been ignored during data analysis. Finally, they should alter the language further to focus on the change in Calb2 levels, not on SGN differentiation.

There are also some questions about the statistics:

With regards to the CI values reported in Fig. 9I:

i) Are they for WT or KO? This needs to be clarified. Why not provide CI for both?

ii) Some CI values appear impossible given the mean values. For instance, when considering analysis of Calb1/Calb2 co-expression, the numbers are as follows (from the legend):

WT Calb2/Calb1: 0.34 {plus minus} 0.023, KO Calb2/Calb1: 0.44 {plus minus} 0.028, 95% CI [-0.16, -0.035], p = 0.0006.

How can the limits of 95% CI both be <0 when the mean proportions in WT and KO are 0.34 and 0.44, respectively?

iii) With regards to the Calb1/Pou4f1 overlap in Fig. 9I: The mean value in KO is less than half that in WT. How can this difference not be statistically significant given such a big difference in means with only modest standard errors or deviations (whatever they are). When I plotted the data myself, there seemed to be little potential for overlapping error bars. If they are getting a p value of 0.5 for this comparison, they should revisit their statistical analysis. I suspect this applies to Calb2/Pou4f1 overlap as well but I didn’t plot the data myself to check.

Minor issues:

1) The stated rationale for certain experiments is not always logical. I advise that the authors reword relevant parts of the text to make sure that the experiment they present in the paper align well with the question they set up. For example, in line 213, they write: “We first wanted to determine the temporal dynamics of SGN debranching during development and did so by examining...”. If this is what they wanted to really do, they’d do live imaging (as noted previously). The experiments show how SGN branch pruning proceeds during early development and how that process is affected in P2rx3 mutants, but do not provide any information into the temporal dynamics of branch loss.

2) Although improved, the discussion is too long and often wanders too far from the core findings. Rather than speculating about possible effects on Type II SGNs or central axons, for instance, there should be a focused discussion of the possible impact of Ca2+ entry. The model of Ca2+ entry through P2rx3 affecting neuronal cytoskeleton directly is fine, but for the sake of considering alternate possibilities, there should also be some discussion of how Ca2+ entry through P2rx3 could affect general activity (spontaneous) in these neurons, which would then affect pruning programs. After all, the evidence for activity-dependent pruning of exuberant processes across the nervous system is wide and strong. And there’s data suggesting spontaneous activity patterns affecting SGN identity, as well as refinement of circuits in the auditory brainstem. Changes in Ca2+ entry could also lead to observed changes in Calb2+ levels, completely independent of the circuit organization or SGN identity or differentiation.

3) Figure 9 Title: Should be reworded considering that only subtle changes in marker expression were observed, which is inadequate to reach any clear conclusion regarding SGN subtype specification.

4) Figure 9 legends and X axis labels in panels I and J: ‘only’ should be added to Calb2, Calb1, Pou4f1 where appropriate to clarify the distinction from bars where double-positives were counted.

## Author Response

Response to the editors and reviewers:

Comment from the editor:

As you will see from the comments below, both reviewers are agreed that your work makes an important advance in the field and that the manuscript has been improved substantially. However, we are agreed that the manuscript would benefit from same additional small changes to the text to clarify the significance of some findings. The comments of both reviewers are appended below. Thank you for submitting your interesting work to eNeuro.

We thank the editor for the kind remarks about our manuscript. We have addressed all of the reviewers’ comments and describe them in point-by-point fashion below. Changes to the main manuscript are shown in blue font.

Reviewer 1:

As far as I am aware, a developmental role for purinergic receptors on the pathfinding and molecular phenotype of spiral ganglion neurons has not previously been studied.

The authors have adequately addressed my comments provided during the first round of review.

We thank Reviewer 1 for the supportive remarks about our manuscript throughout these reviews.

Reviewer 2:

The role of purinergic signaling in cochlear development holds high significance for the field, due both to the known role of ATP in spontaneous activity and the presence of purinergic receptors in developing and mature cochlear cells. This paper reports an extensive, compelling, and well-quantified analysis of the effects of losing one particular purinergic receptor, P2rx3. The data provide new insights into the fine details of how SGN processes branch developmentally and show that P2rx3 plays a role in these events. The authors have done a nice job incorporating the reviewers’ suggestions. The quality of the wholemount stains is outstanding and the analysis they have done, using Sox2-CreER, is both appropriate and illuminating for studying afferent process pruning events in the developing cochlea.

We thank Reviewer 2 for the supportive remarks above, and for the very thoughtful comments below. It is clear that he/she took considerable time to evaluate our manuscript. We are very appreciative of this and the suggestions have been very helpful.

Although the authors softened the language related to the change in Type I SGN subtype markers, some issues still remain, as outlined below. To be clear, the data are fine (though some questions about the statistics need to be addressed - see below). The problem is simply on the overreliance of a few markers to say anything about SGN identity, when the effect reported could have nothing to do with identity at all and this point still needs to made more clearly. This can be dealt with easily with a few more tweaks to the text. Please note that I am pushing this point simply to make sure that readers appreciate the nuances of what this all could mean, not to criticize the data themselves (which are beautiful).

In particular, we have changed how the subtype data in figure 9 are displayed and we have made our interpretation of these data more conservative (with emphasis on increased numbers of Calb2 expressing cells being the primary phenotype). This is described in more detail below where we have mapped out the specific changes. We also agree that the three subtype markers should not be used as any sort of functional readout. To emphasize this point, we have added on page 17, line 417 (in Results) “While the presence of any combination of these factors does not necessarily indicate functional attributes of the SGNs, any changes in their proportions between groups could indicate altered differentiation.” We also added to page 20, line 494 (in Discussion) “...this phenotype possibly represents reduced type I SGN differentiation. Extensive profiling of type I SGN markers and firing characteristics would need to be done to determine if P2rx3 loss leads to any functional changes. As noted below, it is also possible that the increased proportion of Calb2-positive cells in P2rx3-/- cochleae could have resulted from altered Ca2+ fluctuations.”

Major issues:

Figure 9 is still concerning. As indicated in the previous reviews, the authors need to consider the possibility that the phenotype is secondary. They avoid the use of the word specification appropriately, but I don’t think that the word differentiation solves the problem. This point was raised earlier, but let me try to be more explicit this time.

As shown below, we have made numerous changes based on this series of comments. We agree that “specification” or “differentiation” is too strong based on just the immunostaining data. This point was reflected on above. In addition, in the revised manuscript we have now suggested that differentiation may be one of the phenotypic outcomes of the changed profile of subtype markers. The Abstract on page 2, line 55 indicates “These data suggest P2rx3 may be necessary for normal type I SGN differentiation in addition to serving a role in branch refinement.” Page 18, line 436 (Results) indicates “Given this and the increase in numbers of Calb2-positive neurons in the P2rx3-/- cochleae, these data support a model whereby P2rx3 may provide a modest contribution to type I SGN differentiation.” This can also be found on Page 20 starting on line 490 (Discussion): “As shown in Figure 9, we also found that cochleae lacking P2rx3 showed a modest change in how type I SGN subtype markers were represented. In particular, P2rx3-/- cochleae at P30 showed increased numbers of SGNs that were positive for Calb2. Based on previous work showing that type I SGN subtypes become distinguishable by marker expression increasingly over developmental time (Shrestha et al., 2018; Sun et al., 2018), this phenotype possibly represents reduced type I SGN differentiation.”

Here’s the data reported in Figure 9 legend (for 9I):

a. WT Calb2: 0.12 {plus minus} 0.017, KO Calb2: 0.15 {plus minus} 0.02, 95% CI [-0.093, 0.036], p = 0.7908;

b. WT Calb1: 0.37 {plus minus} 0.027, KO Calb1: 0.29 {plus minus} 0.011, 95% CI [0.015, 0.14], p = 0.0088;

c. WT Pou4f1: 0.1 {plus minus} 0.013, KO Pou4f1: 0.09 {plus minus} 0.012, 95% CI [-0.056, 0.073], p = 0.9996

d. WT Calb2/Calb1: 0.34 {plus minus} 0.023, KO Calb2/Calb1: 0.44 {plus minus} 0.028, 95% CI [-0.16, -0.035], p = 0.0006;

e. WT Calb1/Pou4f1: 0.069 {plus minus} 0.012, KO Calb1/Pou4f1: 0.033 {plus minus} 0.0078, 95% CI [-0.029, 0.1], p = 0.581;

f. WT Calb2/Pou4f1: 0.0081 {plus minus} 0.003, KO Calb2/Pou4f1: 0.0033 {plus minus} 0.0016, 95% CI [-0.06, 0.069], p > 0.9999

I calculated the following based on the data above (not counting those overlapping with Pou4f1 because they are relatively small proportions):

Total proportion of cells expressing any Calb2 (exclusive + non-exclusive): 0.12 + 0.34 = 0.46 (WT); 0.15 + 0.44 = 0.59 (KO)

Total proportion of cells expressing any Calb1: 0.37 + 0.34 = 0.71 (WT) and 0.29 + 0.44 = 0.73 (KO)

Thus, the reported change in overlapping Calb2/Calb1 expression can be attributed to ‘expansion’ of Calb2 expression: there is a 28% increase in the proportion of cells deemed to be Calb2+ (exclusive or not). There is no change in proportion of cells expressing Calb1. The difference they report in cells expressing Calb1 only (which is statistically significant) can also result simply from Calb2 proportion being expanded. Therefore, this is the primary phenotype. This is how their results should be reported.

We agree with the reviewer that the change of “Calb2 all” proportion is the primary phenotype. We modified the text in the Results section to reflect this change. We also modified the graph in Figure 9I and J by adding “Calb2 all", “Calb1 all", and “Pou4f1 all” groups and placed them at the beginning of the bar graphs to highlight their importance. This is described in the Results on Page 18, line 425: “As shown in Figure 9I and J, we found that the proportion of SGNs expressing Calb2 (Calb2 all) was increased in the P2rx3-/- cochleae by 11% in the apex and 9.2% in the base. In the apex of P2rx3-/- cochleae, there was a corresponding decrease in SGNs positive only for Calb1, and an increase of SGNs positive for both Calb1 and Calb2 (Figure 9I and J). The base of P2rx3-/- cochleae showed a different scenario: the increase in the entire population of Calb2 expressing cells manifested as a result of an increase in cells positive for Calb2 only.” It is also noted in the Discussion on page 20, line 492: “In particular, P2rx3-/- cochleae at P30 showed increased numbers of SGNs that were positive for Calb2.”

Now, Calb2 is normally expressed in a gradient from Ia to Ic SGNs. Given this, it is critical to report the criteria for assigning cells as Calb2+ vs. Calb2-. As you might imagine, inconsistency in applying cut-offs could easily suggest a proportion change when in fact the only change is the level of Calb2. Especially given the known effect of purinergic signaling on calcium levels, it is easy to envision cells homeostatically increasing Calb2 levels, since Calb2 is a calcium binding protein. I’m not sure I would call this a sign of altered differentiation, but that may be a matter of semantics. In any case, the authors should report how they chose their cut-offs for each of their markers. Did they account for background noise differences in their images in setting thresholds? Did they account for variation in antibody penetration in tissue sections? Were they blind to genotype when they did these analyses? All of these should be stated clearly and the authors should re-analyze their data if these aspects had been ignored during data analysis. Finally, they should alter the language further to focus on the change in Calb2 levels, not on SGN differentiation.

We thank the reviewer for these helpful suggestions. We have now provided a more thorough description in the Methods and Results sections to address all of these issues. We also added a supplementary figure (9-1), which illustrates the quantification method. In the Methods section, starting on page 8, line 199, we added “Type I SGN subtype immunostaining of WT and P2rx3 KO samples was quantified (blinded to genotype) using ImageJ. A maximum intensity projection was made from images captured with a 20x objective (Plan-Apochromat 20x/0.8 M27); triple-immunostaining for Pou4f1, Calb1, Calb2, plus DAPI was visualized in four separate channels (see Figure 9-1 for a detailed illustration). To control for any sample-to-sample variation in background levels, pixel intensities were examined on non-SGNs (see arrowhead in 9-1; possibly Schwann or otic mesenchyme cells). SGNs with strong staining above this value were deemed positive. Pou4f1-positive cells were determined by obvious nuclear staining above background levels. The DAPI channel was used to confirm overlap of Pou4f1 staining with nuclei. We note here that all SGNs show Pou4f1 background staining in the cytoplasm. Anti-Calb2 and anti-Calb1 immunostaining leads to strong signal in both the nucleus and cytoplasm; pixel values of nearby non-SGNs were also used to determine background in these channels. In terms of the workflow, we first determined which SGNs were positive for Pou4f1. We then toggled between channels to determine whether each was also positive for Calb1 or Calb2. Following this, Calb2 and Calb1 single-positive neurons were counted. Finally, overlap between Calb1- and Calb2-positive cells was counted by toggling between channels. Each SGN was annotated along the way to ensure it was not counted twice. Following quantifications, polygons were drawn around SGN bundles to measure the SGN area. SGN density was calculated by dividing total number of cell bodies by the SGN area.”

In the Results section, on page 17, line 421, we added “This method is illustrated in Figure 9-1, where colored circles indicate examples of SGNs counted as positive for one or more marker. The green, salmon, and dark blue circles respectively indicate SGNs positive for Calb1 only, Pou4f1 only, or Calb2 only. The light blue circle highlights an SGN double positive for Calb1 and Calb2; the SGN circled in yellow is double positive for Pou4f1 and Calb1.”

There are also some questions about the statistics:

With regards to the CI values reported in Fig. 9I:

i) Are they for WT or KO? This needs to be clarified. Why not provide CI for both?

ii) Some CI values appear impossible given the mean values. For instance, when considering analysis of Calb1/Calb2 co-expression, the numbers are as follows (from the legend):

WT Calb2/Calb1: 0.34 {plus minus} 0.023, KO Calb2/Calb1: 0.44 {plus minus} 0.028, 95% CI [-0.16, -0.035], p = 0.0006.

How can the limits of 95% CI both be <0 when the mean proportions in WT and KO are 0.34 and 0.44, respectively?

We thank Reviewer #2 for catching these issues and apologize for the confusion. The 95% CI values were reported were not for either WT or KO. We are reporting the 95% CI for the difference of means between the WT and KO groups. In this method, if the range of values spans zero, differences are not statistically significant. Thus, only one 95% CI value was reported for each comparison between the WT and KO and this should have been clearer. We modified the Figure 9 figure legend to offer clarity: “Each 95% CI indicates the difference in means between WT and P2rx3-/- in each group.”

iii) With regards to the Calb1/Pou4f1 overlap in Fig. 9I: The mean value in KO is less than half that in WT. How can this difference not be statistically significant given such a big difference in means with only modest standard errors or deviations (whatever they are). When I plotted the data myself, there seemed to be little potential for overlapping error bars. If they are getting a p value of 0.5 for this comparison, they should revisit their statistical analysis. I suspect this applies to Calb2/Pou4f1 overlap as well but I didn’t plot the data myself to check.

The effort that Reviewer 2 put into this is admirable and we really appreciate it. Indeed, the Calb1/Pou4f1 and Calb2/Pou4f1 groups visually appeared to have differences that one might predict could be statistically significant. But, our calculations do not indicate this and we have left “ns” for not significant. The relevant values are highlighted in yellow in the legend for Figure 9.

During the revision, we realized that a t-test was a more appropriate statistical method for these data. Thus, we updated our statistical tests in the revised version (previously using two-way ANOVA followed by Sidak’s multiple comparisons) by using Welch’s t tests on our updated data after blinded quantification. Our updated results are consistent with our original results.

Minor issues:

1) The stated rationale for certain experiments is not always logical. I advise that the authors reword relevant parts of the text to make sure that the experiment they present in the paper align well with the question they set up. For example, in line 213, they write: “We first wanted to determine the temporal dynamics of SGN debranching during development and did so by examining...”. If this is what they wanted to really do, they’d do live imaging (as noted previously). The experiments show how SGN branch pruning proceeds during early development and how that process is affected in P2rx3 mutants, but do not provide any information into the temporal dynamics of branch loss.

To describe the rationale for the experiments Reviewer #2 is referring to, we have added the following statement to page 9, starting on line 222. Please note that we have removed “dynamics” and used “progression” instead, as we indeed did not perform live imaging. “We first wanted to determine the temporal progression of SGN debranching during development and did so by examining cochleae from mice carrying Sox2CreERT2 (Arnold et al., 2011) and R26RtdTom (Madisen and Zeng, 2010).”

2) Although improved, the discussion is too long and often wanders too far from the core findings. Rather than speculating about possible effects on Type II SGNs or central axons, for instance, there should be a focused discussion of the possible impact of Ca2+ entry.

We really appreciate these thoughtful remarks on the Discussion from Reviewer 2. But, we feel the references to type II SGNs and central axons are important considerations for future work so they are worth including in the discussion. Since we are at around 2200 words in the Discussion, which is well short of the 3,000-word limit afforded by eNeuro, we have elected to keep these short paragraphs.

The model of Ca2+ entry through P2rx3 affecting neuronal cytoskeleton directly is fine, but for the sake of considering alternate possibilities, there should also be some discussion of how Ca2+ entry through P2rx3 could affect general activity (spontaneous) in these neurons, which would then affect pruning programs. After all, the evidence for activity-dependent pruning of exuberant processes across the nervous system is wide and strong. And there’s data suggesting spontaneous activity patterns affecting SGN identity, as well as refinement of circuits in the auditory brainstem.

Changes in Ca2+ entry could also lead to observed changes in Calb2+ levels, completely independent of the circuit organization or SGN identity or differentiation.

We agree that more information on these issues should be added. We have now referenced work from Karl Kandler’s lab for activity-dependent refinement of tonotopic organization. We have added the following text to the existing paragraph on spontaneous activity on page 22, starting on line 539: “There is a large body of evidence that disrupted spontaneous activity patterns impairs refinement of sensory maps (Kandler et al., 2009; Kirkby et al., 2013). In the developing auditory brainstem, the loss of efferent cholinergic neurotransmission alters temporal patterns of spontaneous activity and disrupts topographic refinement of synaptic connections and pruning of axon terminals (Clause et al., 2014). In future work, we will determine the extent to which Ca2+ transients are altered in P2rx3-/- SGNs, which would link P2rx3 and Ca2+ to SGN branch refinement. Additionally, since Calb2 is a Ca2+ binding protein (Schwaller, 2009), changes to the rate or spatial distribution of Ca2+ buffering in P2rx3-/- SGNs might underlie the more widespread distribution of Calb2 or somehow change its program of expression.”

3) Figure 9 Title: Should be reworded considering that only subtle changes in marker expression were observed, which is inadequate to reach any clear conclusion regarding SGN subtype specification.

This figure title (legend and in Results) now reads: P2rx3-/- cochleae show altered proportions of Type I SGN subtype markers

4) Figure 9 legends and X axis labels in panels I and J: ‘only’ should be added to Calb2, Calb1, Pou4f1 where appropriate to clarify the distinction from bars where double-positives were counted.

Add response note here:

Thank you for the suggestion. We added “only” to the Calb2, Calb1, Pou4f1 groups on the X axis in both panels I and J and figure 9 legends.
